# Inferring the kinetics of stochastic gene expression from single-cell RNA-sequencing data

**DOI:** 10.1186/gb-2013-14-1-r7

**Published:** 2013-01-28

**Authors:** Jong Kyoung Kim, John C Marioni

**Affiliations:** 1 European Bioinformatics Institute (EMBL-EBI), Wellcome Trust Genome Sciences Campus, Hinxton, Cambridgeshire, CB10 1SD, UK

**Keywords:** gene regulation, RNA-seq, single-cell, statistics, transcriptional burst

## Abstract

**Background:**

Genetically identical populations of cells grown in the same environmental condition show substantial variability in gene expression profiles. Although single-cell RNA-seq provides an opportunity to explore this phenomenon, statistical methods need to be developed to interpret the variability of gene expression counts.

**Results:**

We develop a statistical framework for studying the kinetics of stochastic gene expression from single-cell RNA-seq data. By applying our model to a single-cell RNA-seq dataset generated by profiling mouse embryonic stem cells, we find that the inferred kinetic parameters are consistent with RNA polymerase II binding and chromatin modifications. Our results suggest that histone modifications affect transcriptional bursting by modulating both burst size and frequency. Furthermore, we show that our model can be used to identify genes with slow promoter kinetics, which are important for probabilistic differentiation of embryonic stem cells.

**Conclusions:**

We conclude that the proposed statistical model provides a flexible and efficient way to investigate the kinetics of transcription.

## Background

RNA-sequencing (RNA-seq) is a recently developed approach that allows an unbiased examination of the transcriptome to be performed using high-throughput DNA sequencing [1-3]. Compared to gene expression microarrays, the previous gold standard for genome-wide quantification of gene expression levels, RNA-seq has some specific advantages: it allows splicing to be assayed in an unbiased manner [4], it better enables the measurement of expression levels over a wide dynamic range [1], and it allows allele-specific expression to be interrogated [5,6].

Until recently, most RNA-sequencing experiments began with a large population of cells (> 10^5^), and, as a result, the gene expression counts obtained can be viewed as an average across that population. However, recent developments in sequencing technology have enabled the use of much smaller volumes of starting material, and several groups have described protocols for assaying the transcriptome of single cells [7-11]. This is vital in many biological contexts, such as early embryonic development and tumor etiology, where it is expected that different cells will have distinctive expression profiles. Furthermore, even in tissues that are typically considered to consist of homogeneous populations of cells, inter-cellular variability in gene expression levels can be considerable. For example, the cells of a genetically identical population grown in the same environment have been shown to display substantial variability in the total number of mRNA molecules that they contain [12-14]. This variability can be partially explained by noting that gene expression levels are regulated by combinatorial interactions between numerous cellular components, where these interactions involve random biochemical reactions [12,13,15].

More generally, single-cell imaging methods (e.g., RNA fluorescence *in situ *hybridization or FISH) have been widely applied to elucidate the principles of gene expression regulation *in vivo *[16]. These studies have observed that: i) gene expression is heterogeneous [12-14]; ii) genes fluctuate between an 'on' and 'off' promoter state and transcripts are produced in bursts [17-19]; and iii) the transition to the 'on' state requires multiple rate-limiting steps that are determined by many sequential interactions between regulators and chromatin, but the transition to the 'off' state can be determined by a single rate-limiting step [16]. Some examples of the stochastic processes that play a role in the transition to the 'on' state are the recruitment of nucleosome remodelers and histone-modifying enzymes by activators, the rate at which RNA polymerase II (PolII) escapes from the core promoter to produce short RNA molecules prior to pausing, and the rate at which PolII leaves pausing and enters productive elongation [15].

One situation where stochastic fluctuation in gene expression levels plays an important role is in the regulation of mouse embryonic stem (ES) cells [14]. Mouse ES cells are derived from the inner cell mass (ICM) or the epiblast of the pre-implantation blastocyst [20], and they can proliferate in the same undifferentiated state indefinitely whilst retaining the ability to differentiate into all adult cell lineages. These two hallmarks of ES cells are conferred by tightly controlled gene regulatory networks [21]. However, growing evidence suggests that the ability of an individual ES cell to differentiate into an adult cell type at a specific time is determined stochastically [14,22]. In particular, the expression levels of key regulatory genes, such as *Nanog, Stella*, and *Rex1*, which are markers of pluripotency, are heterogeneous in ES cells even though the cells are cultured in the same condition [23]. This implies that ES cells exist in a dynamic equilibrium between states that show different propensities for differentiation [22-24].

Here, we develop a statistical framework motivated by a kinetic model for transcriptional bursting to model the biological variability present in single-cell RNA-seq data. The framework derived makes it easy to perform parameter fitting and allows the kinetics of transcription to be investigated. We apply our model to single-cell RNA-sequencing data generated from mouse ES cells and demonstrate that the estimated parameters are consistent with promoter kinetics inferred from RNA polymerase II binding and chromatin state profiles.

## Results

### A kinetic model for stochastic gene expression

**Figure 1:**
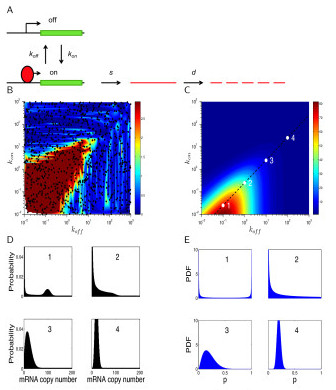
**Poisson-beta model**. **(A) **Schematic of a two-state kinetic model for stochastic gene expression. **(B) **Heat map of the maximum *P *values of two goodness-of-fit tests for Poisson and negative binomial distributions. One thousand combinations of *k*_on _and *k*_off _were uniformly sampled from the log space by fixing *s *to 100. For each combination of the sampled parameters, 1,000 independent samples were generated from the Poisson-beta distribution to evaluate the fit of the data to the Poisson and negative binomial distributions using a bootstrap-based goodness-of-fit test. The colors represent minus log_10_-transformed *P *values and the heat map is interpolated from the scattered data by using a Delaunay triangulation method. **(C) **Heat map of the Fano factor as a function of *k*_on _and *k*_off _with a fixed rate of transcription (*s *= 100). Along the black dashed line fixing the average number of mRNA molecules to 20, the four combinations of *k*_on _and *k*_off _give the varied level of the Fano factor and show different patterns of the variability of the number of mRNA molecules between cells. At point 1 with the highest Fano factor, the transitions between the two promoter states are slow, and the standardized expression level of a gene exhibits a U-shaped distribution, resulting in a bimodal distribution. At point 2, the transition to the inactive state is faster than the transition to the active state, and therefore the mRNA distribution has a long right tail resulting from occasional transcriptional bursts. As *k*_on _and *k*_off _increase at points 3 and 4, transitions between promoter states become fast, resulting in a Poisson-like distribution of the number of mRNA molecules with the Fano factor approaching 1. Note that this plot is similar to a recent figure generated by [25]. **(D) **Representative Poisson-beta distributions from four points in (C), which were computed with the auxiliary variable approach. **(E) **The corresponding beta distributions of *p*.

The standard kinetic model for gene expression assumes that a gene can fluctuate randomly between 'on' and 'off' promoter states, where mRNA can be transcribed only in the 'on' state [16,25] (Figure 1A). If a single rate-limiting step determines the rates of transcription and transitions between the two promoter states [16,17], the fluctuations between the 'on' and 'off' promoter states can be described by a two-state Markov process where *k*_on _is the rate (per unit time) at which a gene becomes active and *k*_off _is the rate (per unit time) at which the gene becomes inactive. Consequently, 1/*k*_off _and 1/*k*_on _describe the average waiting time of a gene in the active and inactive states, respectively, and the (average) fraction of time that a gene spends in the active state is:

(1) $$\bar{p}=\frac{\frac{1}{k_{\text{off}}}}{\frac{1}{k_{\text{off}}}+\frac{1}{k_{\text{on}}}}=\frac{k_{\text{on}}}{k_{\text{off}}+k_{\text{on}}}.$$

Moreover, when the gene is in the active promoter state, it is assumed to be transcribed at a rate, *s*, per unit time and the number of mRNA molecules of the gene is assumed to decay at a rate, *d*, per unit time. Subsequently, transcriptional bursting can be characterized by two parameters: the average number of synthesized mRNA molecules while a gene remains in an active state (burst size or transcriptional efficiency, *s*/*k*_off_) and the frequency at which bursts occur per unit time (burst frequency, *k*_on_) [18,26-28].

Given these four kinetic parameters, a set of differential equations has been derived describing how the number of mRNA molecules of a given gene within a cell, *x*, changes over time (Additional file 1; [17]). The steady state distribution of these equations has been shown to take the form [17-19]:

(2) $$P\left(x|k_{\text{on}},k_{\text{off}},s,d\right)=\frac{{\left(\frac{s}{d}\right)}^{x}e^{-s/d}\;\Gamma \left(\frac{k_{\text{on}}}{d}+x\right)\Gamma \left(\frac{k_{\text{on}}}{d}+\frac{k_{\text{off}}}{d}\right)}{x!\;\Gamma \left(\frac{k_{\text{on}}}{d}+\frac{k_{\text{off}}}{d}+x\right)\Gamma \left(\frac{k_{\text{on}}}{d}\right)}{\;}_{1}F_{1}\left(\frac{k_{\text{off}}}{d},\frac{k_{\text{on}}}{d}+\frac{k_{\text{off}}}{d}+x;\frac{s}{d}\right).$$

As noted previously, the four kinetic parameters are all measured in units of time. However, since the inverse of the decay rate, 1/*d*, denotes the average lifetime of an mRNA molecule, it can be used to normalize the other kinetic parameters so that they are independent of time [16,17]. This is equivalent to setting *d *= 1 in (2), and we do this henceforth.

### A Poisson-beta model

The parameters of the steady-state solution (2) have previously been estimated from observed data using two different approaches. The first approach is to match the first three moments to their empirical values [17]. Although this method is straightforward and computationally efficient, it does not guarantee that the estimates are within the parameter space. To overcome this problem the maximum likelihood estimates of the parameters can be found using a numerical optimization approach [18]. However, the computation of the confluent hypergeometric function, _1_*F*_1_, is difficult, because there is no numerical method for its accurate, fast and reliable computation within all parameter values [29]. Furthermore, when the number of observations is small (less than 100), the maximum likelihood approach sometimes gives unrealistically large estimates of the kinetic parameters [18].

To overcome these limitations, we propose an auxiliary variable approach. Specifically, we let:

(3) $$\begin{array}{c} x|s,p\sim \text{Poisson}\left(sp\right)\\ p|k_{\text{on}},k_{\text{off}}\sim \text{Beta(}k_{\text{on}},k_{\text{off}}\text{)} \end{array}$$

where *p *is an auxiliary variable following a beta distribution. The marginal distribution *P*(*x*|*s, k*_on_, *k*_off_), which is known as the Poisson-beta distribution (PoBe) [30], takes the same form as the steady-state distribution described in equation (2).

Interestingly, the mean of the auxiliary variable *p *is equal to the fraction of time that a gene spends in the active state (1). Further, Smiley and Proulx [31] showed that if a gene's expression level oscillates between 0 and *s*/*d *and the maximum expression level of the gene (*s*/*d*) is set to 1, then given the two-state model, its stationary distribution takes the same form as that of *p*.

Given count measurements from RNA-sequencing data, we assume that the number of reads mapped to a gene is proportional to the expression of the relevant mRNA molecule in the cell under study, and thus the parameters of the kinetic model can be inferred using a Bayesian hierarchical approach, such as a Gibbs sampler.

One of the most significant challenges in applying the kinetic model for gene expression is interpreting the parameters. As noted in a recent review by Munsky *et al. *[25], when *k*_on _and *k*_off _are large the transitions between the promoter states are rapid, resulting in a Poisson or negative binomial-like distribution of the number of mRNA molecules [18,19]. In the context of fitting the kinetic model to real data this corresponds to areas of the parameter space where the three parameters are not identifiable (Method; Figure 1B). By contrast, when *k*_on _and *k*_off _are small, there are relatively few transitions between the two promoter states and the resulting distribution of gene expression molecules between different cells is bimodal - here all three parameters are identifiable (Figure 1B-E).

In practice, to ensure that the parameters are statistically identifiable, we suggest fitting three models (Poisson, negative binomial and Poisson-beta) to each gene before using a goodness-of-fit statistic to determine whether there is evidence that the parameters of the Poisson-beta model can be identified unambiguously (Methods). An alternative approach would be to fit a hierarchical Bayesian model to each gene and to use this to determine the best fitting distribution.

### Assessing the reliability of the Poisson-beta model

Single-cell RNA-sequencing was recently used to assay the transcriptome of 12 mouse ES cells derived from the ICM at embryonic day 3.5 (E3.5) [8]. To explore the transcriptional kinetics of ES cells, we fitted the Poisson-beta model to these data (Methods). Before interpreting the inferred kinetic parameters, it is necessary to: i) account for the high amount of technical variability present in single-cell RNA-seq data; ii) consider whether the parameter estimates are statistically identifiable; and iii) assess whether we can draw meaningful inferences about transcriptional kinetics based on gene expression measurements from 12 cells.

Accurately quantifying the technical variability present in single-cell RNA-seq data is challenging. While experimental approaches vary, most suggest that when replicate libraries are generated from small quantities of RNA (taken from the same, large, population of RNA), the resulting read counts display more technical variability, especially for lowly expressed genes, than is observed in population-based RNA-sequencing analyses [7,10,11]. This is likely due to experimental factors such as the efficiency of the RT step and the PCR amplification when small quantities of starting material are considered [7,10,11]. Some attempts have been made to characterize the technical variation using spike-ins [11] but evidence for the efficacy of such approaches is still limited. Given these challenges and the limitations of current experimental approaches, we instead removed lowly expressed genes that are most likely to display high technical variability [7,10,11]. We considered a gene as lowly expressed if the maximum normalized read count was less than 50. This cutoff was determined using technical replicate data generated using the same protocol applied to the 12 ES cells [8] or oocytes [7] (Additional file 1, Figure S2). Across the set of 18,735 genes that were expressed in at least one cell, 12,551 genes had an expression level above this cutoff, and we fitted the Poisson-beta model separately to each of these genes (Additional file 1, Figure S3).

Using the identifiability criteria outlined in the previous section, we determined that 10,298 (82%) of the 12,551 genes had identifiable parameters at a *P *value threshold of 0.1 (Methods). The genes with non-identifiable parameters could be split into two broad categories (Additional file 1, Figure S3):

1. Genes with relatively large values of *k*_off,*i *_and low values of *k*_on,*i*_. This corresponds to genes that have a low expression count in most cells and high expression in a small number of cells (typically one). When we simulated data from the Poisson-beta model with parameter values in this range (Methods), we found that *k*_on,*i *_was estimated accurately, but that both *k*_off,*i *_and *s_i _*were underestimated (Additional file 1, Figures S6, S11).

2. Genes with large values of *k*_off,*i *_and *k*_on,*i *_(Additional file 1, Figure S3). This set of genes are typically highly expressed (Additional file 1, Figure S12C, F) with a relatively low amount of variability across cells, as evidenced by the large values of *k*_off,*i *_and *k*_on,*i*_. While it is possible that this set of genes do have very fast promoter kinetics, statistically it is impossible to distinguish this from their being permanently in an active state (that is, *k*_off,*i *_is equal to or very close to zero). Hence, it is impossible to interpret either the raw or the derived parameters in this situation (Additional file 1, Figure S12). More generally, this explains why we do not observe many low values of *k*_off,*i *_in our set of parameter estimates. Moreover, it helps to explain some of the identifiability problems that other approaches have encountered when estimating *k*_off,*i*_.

Given these observations, we focus henceforth on the 10,298 genes that have identifiable estimates of the kinetic parameters. However, before going on to make biological inferences based upon these parameters it is first necessary to assess whether any meaningful conclusions can be drawn from fitting the Poisson-beta model to data from only 12 independent ES cells.

To do this, we fitted the Poisson-beta model to data simulated using the estimated parameters by increasing the number of cells from 3 to 100 (Methods). As expected, the correlation between the parameter estimates and the true values improved as the number of cells increased (Additional file 1, Figure S4-S8), with a good agreement when 12 cells were considered (Additional file 1, Figure S6-S8). Our simulations also displayed a tendency to underestimate *s_i_*; the extent of the underestimation decreased as the number of cells increased (Additional file 1, Figure S8). One effect of this is a slight bias in the estimated values of *k*_on,*i *_and *k*_off,*i *_(Additional file 1, Figures S6-S8). This is not unexpected since *s_i _*can be considered to represent the 'maximum' rate of transcription and, especially when the number of cells is small, a cell where a gene is expressed at this 'maximal' value will not be simulated. Nevertheless, our simulations do provide confidence in the fit of the Poisson-beta model when a moderate number of cells (greater than or equal to 12) are considered. However, it is important to acknowledge that drawing strong biological inferences about the kinetic parameters of individual genes from only 12 cells is difficult - hence, in what follows we consider properties of sets of genes with specific values of the kinetic parameters. This will also help mitigate any effect that technical noise in the measurement of gene expression levels will have upon our interpretation of the data.

### Transcriptional kinetics of mouse ES cells

To explore how the kinetic parameters provide information about the regulation of transcription we utilized independent information collected from v6.5 mouse ES cells (derived from the ICM at E3.5) on RNA polymerase II (PolII) occupancy and various histone modifications [32-34]. At the global level, the rates of transcription and gene activation are strongly correlated with the average expression level while the rate of gene inactivation displays a more modest correlation (Additional file 1, Figure S13).

**Figure 2:**
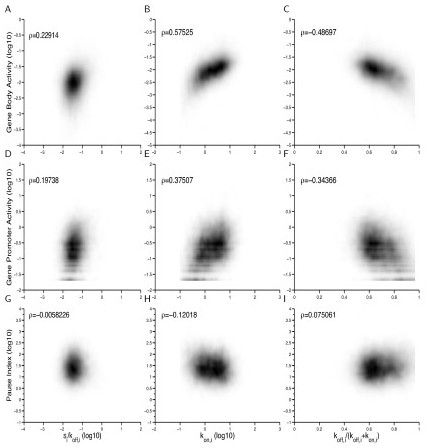
**Correlation of transcriptional kinetics with RNA polymerase II binding in mouse ES cells**. In the left panel, burst size (or transcriptional efficiency) is plotted on the *x*-axis. In the middle panel, burst frequency is plotted on the *x*-axis. In the right panel, the average fraction of time that a gene spends in the inactive state is plotted on the *x*-axis. In all the panels, the following are plotted on the y-axis: the gene body activity **(A)**-**(C)**, the gene promoter activity **(D)**-**(F)**, and the pause index **(G)**-**(I)**. Each point represents one identifiable gene with a normalized read count greater than 50 in at least one cell. ρ is the Spearman correlation coefficient.

As expected, we observed that PolII occupancy was positively correlated with the average expression level (Additional file 1, Figure S14) and burst frequency and, less strongly, with burst size (Figure 2). This is true irrespective of whether PolII levels are calculated in the gene body (*P *< 10^-16 ^by the Spearman rank test) or in the promoter region (*P *< 10^-16^) although the correlation was noticeably higher in the gene body comparison (Figure 2, S14 in Additional file 1). However, when we examined the relationship between burst size and the pause index, defined as the ratio of PolII occupancy in the promoter compared to the gene body, we observed no correlation (*P *= 0.5558; Figure 2G). Moreover, although we found that both burst frequency and the average proportion of time a gene is transcriptionally inactive were significantly correlated with the pause index (*P *< 10^-16 ^for *k*_on,*i*_; P < 10^-13 ^for *k*_off,*i *_/ (*k*_off,*i *_*+ k*_on,*i*_); Figure 2H-I), the correlation is low in both cases, providing only very weak evidence that PolII pausing is associated with burst frequency. A more stringent cutoff that filtered out the lowly expressed genes did not change the results (Additional file 1, Figure S15).

**Figure 3:**
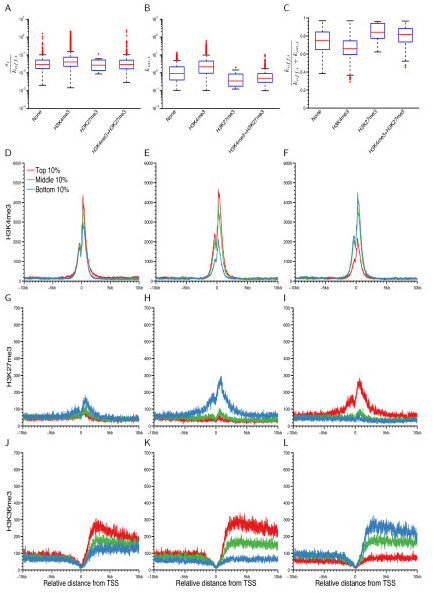
**Correlation of transcriptional kinetics with histone modifications in mouse ES cells**. **(A)**-**(C) **Box plots that compare burst size (A), burst frequency (B), and the average fraction of time that a gene spends in the inactive state (C) in the four groups. Given the annotated chromatin state of the two histone modifications by [33], we classified all expressed genes with a normalized read count greater than 50 in at least one cell that have chromatin state annotations into four groups: H3K4me3 only (*n *= 6,291), H3K27me3 only (*n *= 10), H3K4me3 + H3K27me3 (*n *= 630) and none (*n *= 492). The H3K4me3 group is significantly different from the others except the H3K27me3 group: *P *< 10^-16 ^for *s_i_*/*k*_on,*i*_, *P *< 10^-16 ^for *k*_on,*i*_, *P *< 10^-16 ^for *k*_off,*i*_/(*k*_off,*i *_+ *k*_on,*i*_), by the Mann-Whitney U-test. Due to the small number of samples of the H3K27me3 group, the H3K4me3 group is less significantly different from the H3K27me3 group: *P *= 0.0486 for *s_i_*/*k*_on,*i*_, *P *= 2.73 × 10^-5 ^for *k*_on,*i*_, *P *= 8.12 × 10^-5 ^for *k*_off,*i*_/(*k*_off,*i *_+ *k*_on,*i*_). In each box plot, the central red line indicates the median value, the top and bottom edges of the box are the 75th (*q*3) and 25th (*q*1) percentiles, and the ends of the whiskers denote *q*3 + 1.5(*q*3 - *q*1) and *q*1 - 1.5(*q*3 *q*1). **(D)**-**(L) **The profiles of H3K4me3, H3K27me3, and H3K36me3 ChIP-seq reads mapped near TSSs (Transcription Start Sites) are shown for genes with values of the three kinetic quantities in the top 10% (red), middle 10% (green), and bottom 10% (blue) of the relevant distribution. The *y*-axis is the total number of reads mapped to each position.

Histone modifications can alter chromatin structure, thereby affecting the regulation of gene expression levels [35]. Two of the most widely studied modifications, H3K4me3, which is associated with active promoters, and H3K27me3, which is associated with genes that have repressed expression levels, have previously been positively and negatively correlated with gene expression levels [32]. Our estimated kinetic parameters are consistent with these observations (see figure legend for statistical details, Figure 3A-I, S16 in Additional file 1): genes with a H3K4me3 modification have significantly higher rates of transcriptional bursting and frequency than genes with no modification or those with a H3K27me3 modification. The third histone mark, H3K36me3, which is linked to transcriptional elongation and is enriched over the gene body region [32], was strongly associated with both burst frequency and the fraction of time that a gene spends in the inactive state but more weakly with burst size (Figure 3J-L).

**Figure 4:**
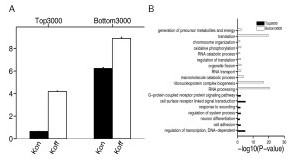
**Functional enrichment of noisy genes showing distinctive bursting patterns of gene expression in mouse ES cells**. **(A) **The mean of *k*_on,*i *_and *k*_off,*i *_(± S.E.) for the top and bottom 3,000 genes sorted by *k*_off,*i*_/ *k*_on,*i*_. (S.E. = standard error). **(B) **The Benjamini corrected *P *values of the 18 GO terms enriched in the top or bottom 3,000 genes. GO, gene oncology.

Finally, we used gene ontology (GO) analysis [36] to interrogate the set of genes that showed characteristics associated with transcriptional bursting (that is, a low value of *k*_on,*i *_and a relatively high value of *k*_off,*i*_). To do this, we sorted all 10,298 genes in descending order according to the ratio of *k*_off,*i *_to *k*_on,*i*_, and considered the top 3,000 genes from this list. As a control, we chose the bottom 3,000 genes from the sorted list (Figure 4A). We found that genes with characteristics of rapid transcriptional bursting were associated with 'cell adhesion' (Figure 4B), consistent with previous reports that many tissue-specific cell adhesion molecules are expressed in mouse ES cells [37] and show cell-to-cell variation in expression in mouse ES cell colonies [38]. Interestingly, the gene ontology category 'neural differentiation' was also enriched (Figure 4B), providing some support for previous studies that suggested that neural fate is chosen in a stochastic way [14,39]. Conversely, the least varying set was enriched with genes associated with the maintenance of basic cellular function (Figure 4B), suggesting that eukaryotic cells have evolved to reduce the transcriptional noise of housekeeping genes for the phenotypic stability of basic cellular functions [14].

## Discussion

The Poisson-beta model provides a convenient statistical framework for modeling single-cell RNA-seq data and for studying the kinetics of stochastic gene expression. Since the kinetic parameters of individual genes inferred from the small number of cells are likely to be noisy and may be influenced by technical variability, we focused on the summary properties of genes. Importantly, we confirmed that the kinetic parameters derived from the Poisson-beta model are consistent with PolII binding and chromatin modifications using single-cell RNA-seq data generated from mouse ES cells. Our results suggest that the chromatin state of genes, defined by H3K4me3, H3K27me3 and H3K36me3 modifications, affects transcriptional bursting by modulating both burst size and frequency, consistent with a recent study that suggested chromosomal location affected these kinetic characteristics [40].

However, while our model has clear advantages, it also has a number of limitations. First, in this manuscript, we do not address the modeling of the technical variability directly, primarily because our understanding of how different experimental characteristics (RT efficiency, PCR amplification, etc.) might contribute to the noise is very limited. Instead, we focus only on genes that are moderately to highly expressed, since previous single-cell RNA-seq studies have shown that such genes display less technical variability. However, as our understanding of the technical variability inherent to single-cell RNA-seq increases, it will be important to adapt the model presented herein.

Second, in common with most other biochemical models of gene expression, we assume that the rate of transitions to the 'on' state is governed by a single rate-limiting step. While this assumption facilitates the derivation of a closed-form solution for the master equations, and thus the implementation of the Poisson-beta model described in this paper, in higher eukaryotes activation requires many sequential steps [15]. However, the limited experimental data about the relative contribution of the different steps justifies the simplified model presented herein.

Third, the three kinetic parameters are currently measured in units of 'per mRNA average lifetime' since they are normalized by the decay rate. To estimate them in units of 'per second', we should directly measure the decay rates of all genes. This can be done by metabolic labeling of RNA with 4-thiouridine coupled with massively parallel sequencing [41]. Another improvement would be to measure the number of mRNA molecules directly rather than using the number of reads as a surrogate, which can be done by accurate digital quantification of transcriptome via digital RNA-seq [42].

Finally, our model assumes that the transition times and kinetic parameters are identical for the two alleles of each gene. A recent study established that 39% to 51% of heterozygous loci show allele-specific expression when expression patterns are measured in single cells of a two-cell embryo [43]. This suggests that the kinetic parameters and transition times of the underlying Markov chain might differ significantly between the two alleles of a gene. Further, Miyanari *et al. *[44] showed that *Nanog *is largely expressed from a single allele in ES cells and can transition between alleles randomly. Such variability can be incorporated into our model by measuring the expression of each allele independently (for example by using MMSEQ [45]), and using these measures as the input to the model. However, the mouse ES cell data we analyzed were generated from an inbred population of mice (C57BL/6J) and, as a result, we could not apply this approach. Examining allele-specific variation using the Poisson-beta model provides an interesting avenue for future research.

## Conclusions

To summarize, as the single-cell field progresses towards analyzing the transcriptome of large numbers of individual cells in parallel, it will become increasingly important to develop statistical methods that accurately model stochastic gene expression. In this context, we anticipate that the Poisson-beta model presented here, and other similar approaches, will be vital in maximizing the amount of biological insight that can be obtained from these data.

## Materials and methods

### Properties of the steady-state solution of the master equation

The steady-state solution of the chemical master equations can be written as a beta convolution of Poisson random variables (equation (3)). The mean and variance of the Poisson-beta distribution with these parameters are given by:

$$\begin{array}{c} E\left[x\right]=\frac{k_{\text{on}}}{k_{\text{on}}+k_{\text{off}}}s\\ \text{Var[}x\text{]}=E\left[x\right]+\frac{k_{\text{on}}k_{\text{off}}}{{\left(k_{\text{on}}+k_{\text{off}}\right)}^{2}}\frac{s^{2}}{\left(k_{\text{on}}+k_{\text{off}}+1\right)}. \end{array}$$

The squared coefficient of variation, η^2^, and the Fano factor, φ, are given by:

$$\begin{array}{c} \eta^{2}=\frac{k_{\text{on}}+{\text{k}}_{\text{off}}}{{\text{k}}_{\text{on}}\text{s}}+\frac{k_{\text{off}}}{k_{\text{on}}\left(k_{\text{on}}+{\text{k}}_{\text{off}}+1\right)}\\ \phi =1+\frac{sk_{\text{off}}}{\left(k_{\text{on}}+{\text{k}}_{\text{off}}\right)\left(k_{\text{on}}+{\text{k}}_{\text{off}}+1\right)}. \end{array}$$

### A hierarchical Bayesian model

To estimate the parameters of the Poisson-beta distribution, we utilized a hierarchical Bayesian model, which can be described as:

1. Draw *s_i _*for gene *i *from a gamma distribution:

$$s_{i}\sim \text{Gamma }(s_{i}|\alpha_{s_{i}},\beta_{s_{i}})$$

2. Draw *k*_on,*i *_for gene *i *from a gamma distribution:

$${k_{\text{on,}}}_{i}\sim \text{Gamma }({k_{\text{on,}}}_{i}|\alpha_{k_{\text{on}}{,}_{i}},\beta_{k_{\text{on}}{,}_{i}})$$

3. Draw *k*_off,*i *_for gene *i *from a gamma distribution

$${k_{\text{off,}}}_{i}\sim \text{Gamma }({k_{\text{off,}}}_{i}|\alpha_{k_{\text{off}}{,}_{i}},\beta_{k_{\text{off}}{,}_{i}})$$

4. Draw *p_ij _*for gene *i *and cell *j *from a beta distribution

$$p_{ij}\sim \text{Beta }(p_{ij}|k_{\text{on},i},k_{\text{off},i})$$

5. Draw *x_ij _*from a Poisson distribution

$$x_{ij}\sim \text{Poisson}(x_{ij}\text{|}{\text{t}}_{i}t_{j}s_{i}p_{ij})$$

where *t_i _*is the length of gene *i *(the length of the transcripts measured in bp) and *t_j _*is the normalization factor for cell *j*. We used the scale normalization method of [46] to estimate the normalization factor for each cell. We make an implicit assumption that the number of reads is proportional to the number of mRNA molecules present in a cell.

The graphical model representing this generative process is shown in Additional file 1, Figure S1.

### Learning by collapsed Gibbs sampling

Let $X=\{x_{ij}\}$ be a set of observed read counts, and $P=\left\{p_{ij}\right\}$ be a set of *p_ij_*. We treat the top-level variables, shown in the graphical model in Additional file 1, Figure S1, $\Psi =\left\{\alpha_{k_{\text{on},i}},\beta_{k_{\text{on},i}},\alpha_{k_{\text{off},i}},\beta_{k_{\text{off},i}},\alpha_{s_{i}},\beta_{s_{i}}\right\}$, as fixed hyperparameters. We derive a collapsed Gibbs sampler to infer all unknown variables $\Theta =\left\{P,\left\{k_{\text{on},i}\right\},\left\{k_{\text{off},i}\right\},\left\{s_{i}\right\}\right\}$ given X . In the following, a subscript with a minus sign that is attached to a set of variables means that the variables indexed by the subscript are excluded from the set.

The full conditional distributions of *s_i_, p_ij_, k*_on,*i*_, and *k*_off,*i *_are non-standard univariate. To sample the variables from their full conditional distributions, we use slice sampling [47]. For completeness, the full conditional distributions are given below:

1. Sampling *p_ij_*

$$P\left(p_{ij}|P_{-ij},X,\Theta \backslash P,\Psi \right)\propto P\left(p_{ij}|k_{\text{on},i},k_{\text{off},i}\right)P\left(x_{ij}|s_{i},p_{ij}\right)$$

2. Sampling *k*_on,*i*_

$$P\left(k_{\text{on},i}|\left\{k_{\text{on},-i}\right\},X,\Theta \backslash \left\{k_{\text{on},i}\right\},\Psi \right)\propto P\left(k_{\text{on},i}|\alpha_{k_{\text{on},i}},\beta_{k_{\text{on},i}}\right)\overset{J}{\underset{j=1}{\prod }}P\left(p_{ij}|k_{\text{on},i},k_{\text{off},i}\right)$$

3. Sampling *k*_off,*i*_

$$P\left(k_{\text{off},i}|\left\{k_{\text{off},-i}\right\},X,\Theta \backslash \left\{k_{\text{off},i}\right\},\Psi \right)\propto P\left(k_{\text{off},i}|\alpha_{k_{\text{off},i}},\beta_{k_{o\text{off,}i}}\right)\overset{J}{\underset{j=1}{\prod }}P\left(p_{ij}|k_{\text{on},i},k_{\text{off},i}\right)$$

4. Sampling *s_i_*

$$P\left(s_{i}|\left\{s_{-i}\right\},X,\Theta \backslash \left\{s_{i}\right\},\Psi \right)\propto P\left(s_{i}|\alpha_{s_{i}},\beta_{s_{i}}\right)\overset{J}{\underset{j=1}{\prod }}P\left(x_{ij}|s_{i},p_{ij}\right)$$

The log posterior probability, which can be used to monitor the convergence of the Gibbs sampler, is given by

ln P(Θ|X,A)=∑i=1N∑j=1Jxijln titjsipij-titjsipij-ln xij!+∑i=1N∑j=1Jln Γ(kon,i+koff,i)Γ(kon,i)Γ(koff,i)+(kon,i-1)ln pij+(koff,i-1)ln (1-pij)-siβsi+(αsi-1)ln si-αsiln βsi-ln Γ(αsi)-kon,iβkon,i+(αkon,i-1)ln kon,i-αkon,iln βkon,i-ln Γ(αkon,i)-koff,iβkoff,i+(αkoff,i-1)ln koff,i-αkoff,iln βkoff,i-ln Γ(αkoff,i).

For the hyperparameters, we used the following settings: $\alpha_{s_{i}}=1$, $\beta_{s_{i}}=\max_{j}x_{ij}$, $\alpha_{k_{\text{on},i}}=1$, $\beta_{k_{\text{on},i}}=100$, $\alpha_{k_{\text{off},i}}=1$ and $\beta_{k_{\text{off},i}}=100.$ We chose the empirical Bayes prior on *s_i _*so that it becomes almost uniform across all realistic ranges for the parameter. The priors on *k*_on,*i *_and *k*_off,*i *_were chosen to place substantial probability across the identifiable parameter space. When we used different priors on *k*_on,*i *_and *k*_off,*i *_($\alpha_{k_{\text{on},i}}=1$, $\beta_{k_{\text{on},i}}=10,000$, $\alpha_{k_{\text{off},i}}=1$, $\beta_{k_{\text{off},i}}=10,000$ for more diffuse priors and $\alpha_{k_{\text{on},i}}=1$, $\beta_{k_{\text{on},i}}=10$, $\alpha_{k_{\text{off},i}}=1$, $\beta_{k_{\text{off},i}}=10$ for more concentrated priors), the inferred kinetic parameters remained similar, except for the large values of the parameters that were penalized by the concentrated prior. These results suggest that our model is relatively insensitive to the choice of priors (Additional file 1, Figure S9, S10).

### Bootstrap-based goodness-of-fit test

To assess whether a set of observations generated from a Poisson-beta distribution follows a Poisson or negative binomial distribution, we used the parametric bootstrap for goodness-of-fit testing [48]. We first generated *n *independent samples *X*_1_,..., *X_n _*from the Poisson-beta distribution with given parameters using the auxiliary variable representation. We then fitted these *n *simulated samples to the Poisson and negative binomial distributions using a maximum likelihood approach. The MATLAB function 'nbinfit' was used to compute the maximum likelihood estimates of the parameters of the negative binomial distribution. Based on the maximum likelihood estimates of the Poisson or negative binomial distribution, $\theta_{n}^{\text{dist}}\left(\text{dist}\in \left\{\text{Poisson},\text{NB}\right\}\right)$, we computed the Kolmogorov-Smirnov (KS) test statistic ${\text{KS}}_{n}^{\text{dist}}$ such that

$${\text{KS}}_{n}^{\text{dist}}=\max_{i}|F_{n}\left(X_{i}\right)-F_{\theta_{n}^{\text{dist}}}\left(X_{i}\right)|$$

where *F_n _*is the empirical distribution function for the *n *independent samples and $F_{\theta_{n}^{\text{dist}}}$ is the cumulative distribution function of the Poisson or negative binomial distribution with the maximum likelihood estimates $\theta_{n}^{\text{dist}}$. To evaluate the bootstrap *P *value, we repeated the following steps from *k *= 1 to *k *= *B*:

1. Given the maximum likelihood estimates $\theta_{n}^{\text{dist}}$, generate *n *bootstrap samples $X_{1,k}^{*},\ldots ,X_{n,k}^{*}$ from $F_{\theta_{n}^{\text{dist}}}\left(\text{dist}\in \left\{\text{Poisson},\text{NB}\right\}\right)$.

2. Compute the maximum likelihood estimates $\theta_{n,k}^{*,\text{dist}}$ from the bootstrap samples.

3. Estimate the empirical distribution function of the bootstrap samples

$$F_{n,k}^{*}\left(x\right)=\frac{1}{n}\overset{n}{\underset{i=1}{\sum }}1\left(X_{i,k}^{*}\leq x\right).$$

4. Compute the KS test statistic ${\text{KS}}_{n,k}^{*,\text{dist}}$ such that

$${\text{KS}}_{n,k}^{*,\text{dist}}=\max_{i}|F_{n,k}^{*}\left(X_{i,k}^{*}\right)-F_{\theta_{n,k}^{*,\text{dist}}}\left(X_{i,k}^{*}\right)|.$$

Finally, the bootstrap *P *value is given by

$$\frac{1}{B}\overset{B}{\underset{k=1}{\sum }}1\left({\text{KS}}_{n,k}^{*,\text{dist}}>{\text{KS}}_{n}^{\text{dist}}\right).$$

For this study, we set *n *= 1,000 and *B *= 1,000.

### Estimating the kinetic parameters from synthetic data

Given the posterior means of *s_i_, k*_on,*i *_and *k*_off,*i *_for each gene, we generated 3, 6, 12, 20 or 100 independent samples from the Poisson-beta distribution. We run the Gibbs sampling algorithm by setting the total number of Gibbs iterations to 10,000, and computed the posterior means by discarding the first half of the samples in each chain as a burn-in period.

### Gene activity and pause index for GRO-Seq

To quantify PolII activity at the promoters and gene body regions, we defined three measures based on the number of mapped reads of GRO-Seq data [49]. First, gene body activity is defined as *N*/*L *where *N *is the number of GRO-Seq reads mapped from +1 kb of the transcription start site to the end of a gene, and *L *is the length of the region. Second, gene promoter activity is defined as the maximum count of reads in a 50 bp window, where we took the maximum among all the windows within the ± 1 kb region of transcription start sites. Finally, we defined the pause index as the ratio of the gene promoter activity (divided by 50) to the gene body activity.

### Gene ontology analysis using DAVID

To examine whether particular classes of GO biological processes (GOTERM_BP_FAT) are enriched in the top or bottom 3,000 genes sorted by *k*_off,*i*_*/ k*_on,*i*_, we used the DAVID functional annotation clustering tool (the classification stringency was set to 'medium') [36]. By setting up the 10,298 genes as a background, we chose a representative GO term from each annotation cluster with the Benjamini-corrected *P *value less than 0.05, providing 18 GO terms in total. The results are in Additional file 2, Table S1 (top 3,000) and Additional file 3, Table S2 (bottom 3,000).

### Code availability

The MATLAB source code and a compiled version of the same are available in Additional file 4.

## Abbreviations

bp: base pair; ES cell: embryonic stem cell; GO: gene ontology; ICM: inner cell mass; PCR: polymerase chain reaction; PoBe: Poisson-beta; RNA FISH: RNA fluorescence *in situ *hybridization; RNA PolII: RNA polymerase II; RT: reverse transcriptase.

## Competing interests

We declare no conflict of interests.

## Authors' contributions

JKK performed all analysis and wrote all code. JKK and JCM wrote the manuscript and conceived the project. All authors read and approved the final manuscript.

## Supplementary Material

Additional file 1 **Supplemental methods and list of supplemental figures**. Click here for file

Additional file 2 **Table S1**. Click here for file

Additional file 3 **Table S2**. Click here for file

Additional file 4 **MATLAB source files and the compiled version of the same**. Click here for file

## References

[B1] Mortazavi A Williams BA McCue K Schaeffer L Wold B Mapping and quantifying mammalian transcriptomes by RNA-Seq. Nature Methods 2008 5 621 628 10.1038/nmeth.1226 18516045 PMC13303166

[B2] Marioni JC Mason CE Mane SM Stephens M Gilad Y RNA-seq: an assessment of technical reproducibility and comparison with gene expression arrays. Genome Research 2008 18 1509 1517 10.1101/gr.079558.108 18550803 PMC2527709

[B3] Wang Z Gerstein M Snyder M RNA-Seq: a revolutionary tool for transcriptomics. Nature Reviews Genetics 2009 10 57 63 10.1038/nrg2484 19015660 PMC2949280

[B4] Wang ET Sandberg R Luo S Khrebtukova I Zhang L Mayr C Kingsmore SF Schroth GP Burge CB Alternative isoform regulation in human tissue transcriptomes. Nature 2008 456 470 476 10.1038/nature07509 18978772 PMC2593745

[B5] Degner JF Marioni JC Pai AA Pickrell JK Nkadori E Gilad Y Pritchard JK Effect of read-mapping biases on detecting allele-specific expression from RNA-sequencing data. Bioinformatics 2009 25 3207 3212 10.1093/bioinformatics/btp579 19808877 PMC2788925

[B6] Zhang K Li JB Gao Y Egli D Xie B Deng J Li Z Lee JH Aach J Leproust EM Eggan K Church GM Digital RNA allelotyping reveals tissue-specific and allele-specific gene expression in human. Nature Methods 2009 6 613 618 10.1038/nmeth.1357 19620972 PMC2742772

[B7] Tang F Barbacioru C Wang Y Nordman E Lee C Xu N Wang X Bodeau J Tuch BB Siddiqui A Lao K Surani MA mRNA-Seq whole-transcriptome analysis of a single cell. Nature Methods 2009 6 377 382 10.1038/nmeth.1315 19349980

[B8] Tang F Barbacioru C Bao S Lee C Nordman E Wang X Lao K Surani MA Tracing the derivation of embryonic stem cells from the inner cell mass by single-cell RNA-Seq analysis. Cell Stem Cell 2010 6 468 478 10.1016/j.stem.2010.03.015 20452321 PMC2954317

[B9] Tang F Lao K Surani MA Development and applications of single-cell transcriptome analysis. Nature Methods 2011 8 S6 S11 10.1038/nchembio.740 21451510 PMC3408593

[B10] Islam S Kjällquist U Moliner A Zajac P Fan JB Lonnerberg P Linnarsson S Characterization of the single-cell transcriptional landscape by highly multiplex RNA-seq. Genome Research 2011 21 1160 1167 10.1101/gr.110882.110 21543516 PMC3129258

[B11] Ramskold D Luo S Wang YC Li R Deng Q Faridani OR Daniels GA Khrebtukova I Loring JF Laurent LC Schroth GP Sandberg R Full-length mRNA-Seq from single-cell levels of RNA and individual circulating tumor cells. Nature Biotechnology 2012 30 777 782 10.1038/nbt.2282 22820318 PMC3467340

[B12] Kaern M Elston TC Blake WJ Collins JJ Stochasticity in gene expression: from theories to phenotypes. Nature Reviews Genetics 2005 6 451 464 10.1038/nrg1615 15883588

[B13] Raj A van Oudenaarden A Nature, nurture, or chances: stochastic gene expression and its consequences. Cell 2008 135 216 226 10.1016/j.cell.2008.09.050 18957198 PMC3118044

[B14] Eldar A Elowitz MB Functional roles for noise in genetic circuits. Nature 2010 467 167 173 10.1038/nature09326 20829787 PMC4100692

[B15] Fuda NJ Ardehali MB Lis JT Defining mechanisms that regulate RNA polymerase II transcription *in vivo*. Nature 2010 461 186 192 19741698 10.1038/nature08449 PMC2833331

[B16] Larson DR What do expression dynamics tell us about the mechanism of transcription? Current Opinion in Genetics & Development 2011 21 591 599 10.1016/j.gde.2011.07.010 21862317 PMC3475196

[B17] Peccoud J Ycart B Markovian modelling of gene product synthesis. Theoretical Population Biology 1995 48 222 234 10.1006/tpbi.1995.1027

[B18] Raj A Peskin CS Tranchin D Vargas DY Tyagi S Stochastic mRNA synthesis in mammalian cells. PLoS Biology 2006 4 e309 10.1371/journal.pbio.0040309 17048983 PMC1563489

[B19] Shahrezaei V Swain PS Analytical distributions for stochastic gene expression. Proceedings of the National Academy of Sciences, USA 2008 105 17256 17261 10.1073/pnas.0803850105 PMC2582303 18988743

[B20] Young RA Control of the embryonic stem cell state. Cell 2011 144 940 954 10.1016/j.cell.2011.01.032 21414485 PMC3099475

[B21] Huang S Cell lineage determination in state space: a systems view brings flexibility to dogmatic canonical rules. PLoS Biology 2010 8 e1000380 10.1371/journal.pbio.1000380 20520792 PMC2876052

[B22] Martinez AA Brickman JM Gene expression heterogeneities in embryonic stem cell populations: origin and function. Current Opinion in Cell Biology 2011 23 1 7 10.1016/j.ceb.2010.12.003 21982544

[B23] Silva J Smith A Capturing Pluripotency. Cell 2008 132 532 536 10.1016/j.cell.2008.02.006 18295569 PMC2427053

[B24] Canham MA Sharov AA Ko MS Brickman JM Functional heterogeneity of embryonic stem cells revealed through translational amplification of an early endodermal transcript. PLoS Biology 2010 8 e1000379 10.1371/journal.pbio.1000379 20520791 PMC2876051

[B25] Munsky B Neuert G van Oudenaarden A Using gene expression noise to understand gene regulation. Science 2012 336 183 187 10.1126/science.1216379 22499939 PMC3358231

[B26] Skupsky R Burnett JC Foley JE Schaffer DV Arkin AP HIV promoter integration site primarily modulates transcriptional burst size rather than frequency. PLoS Computational Biology 2010 6 e1000952 10.1371/journal.pcbi.1000952 20941390 PMC2947985

[B27] Batenchuk C St-Pierre S Tepliakova L Adiga S Szuto A Kabbani N Bell JC Baetz K Kaern M Chromosomal position effects are linked to sir2-mediated variation in transcriptional burst size. Biophysical Journal 2011 100 L56 L58 10.1016/j.bpj.2011.04.021 21575565 PMC3093560

[B28] Miller-Jensen K Dey SS Schaffer DV Arkin AP Varying virulence: epigenetic control of expression noise and disease processes. Trends in Biotechnology 2011 29 517 525 10.1016/j.tibtech.2011.05.004 21700350

[B29] Muller KE Computing the confluent hypergeometric function, *M*(*a, b, x*). Numerishe Mathematik 2001 90 179 196 10.1007/s002110100285

[B30] Johnson NL Kemp AW Kotz S Univariate discrete distributions 2005 Wiley

[B31] Smiley MW Proulx SR Gene expression dynamics in randomly varying environments. Journal of Mathematical Biology 2010 61 231 251 10.1007/s00285-009-0298-z 19756606

[B32] Mikkelsen TS Ku M Jaffe DB Issac B Lieberman E Giannoukos G Alvarez P Brockman W Kim TK Koche RP Lee W Mendenhall E O'Donovan A Presser A Russ C Xie X Meissner A Wernig M Jaenisch R Nusbaum C Lander ES Bernstein BE Genome-wide maps of chromatin state in pluripotent and lineage-committed cells. Nature 2007 448 553 560 10.1038/nature06008 17603471 PMC2921165

[B33] Ku M Koche RP Rheinbay E Mendenhall EM Endoh M Mikkelsen TS Presser A Nusbaum C Xie X Chi AS Adli M Kasif S Ptaszek LM Cowan CA Lander ES Koseki H Bernstein BE Genomewide analysis of PRC1 and PRC2 occupancy identifies two classes of bivalent domains. PLoS Genetics 2008 4 e1000242 10.1371/journal.pgen.1000242 18974828 PMC2567431

[B34] Min IM Waterfall JJ Core LJ Munroe RJ Schimenti J Lis JT Regulating RNA polymerase pausing and transcription elongation in embryonic stem cells. Genes & Development 2011 25 742 754 10.1101/gad.2005511 21460038 PMC3070936

[B35] Suganuma T Workman JL Signals and combinatorial functions of histone modifications. Annual Review of Biochemistry 2011 80 473 499 10.1146/annurev-biochem-061809-175347 21529160

[B36] Huang DW Sherman BT Lempicki RA Systematic and integrative analysis of large gene lists using DAVID bioinformatics resources. Nature Protocols 2009 4 44 57 19131956 10.1038/nprot.2008.211

[B37] Gu B Zhang J Wang W Mo L Zhou Y Chen L Liu Y Zhang M Global expression of cell surface proteins in embryonic stem cells. PLoS ONE 2010 5 e15795 10.1371/journal.pone.0015795 21209962 PMC3012103

[B38] Cui L Johkura K Yue F Ogiwara N Okouchi Y Asanuma K Sasaki K Spatial distribution and initial changes of SSEA-1 and other cell adhesion-related molecules on mouse embryonic stem cells before and during differentiation. Journal of Histochemistry & Cytochemistry 2004 52 1447 1457 10.1369/jhc.3A6241.2004 15505339 PMC3957812

[B39] Hemmati-Brivanlou A Melton D Vertebrate embryonic cells will become nerve cells unless told otherwise. Cell 1997 88 13 17 10.1016/S0092-8674(00)81853-X 9019398

[B40] Dar RD Razooky BS Singh A Trimeloni TV McCollum JM Cox CD Simpson ML Weinberger LS Transcriptional burst frequency and burst size are equally modulated across the human genome. Proceedings of the National Academy of Sciences, USA 2012 109 17454 17459 10.1073/pnas.1213530109 PMC3491463 23064634

[B41] Rabani M Levin JZ Fan L Adiconis X Raychowdhury R Garber M Gnirke A Nusbaum C Hacohen N Friedman N Amit I Regev A Metabolic labeling of RNA uncovers principles of RNA production and degradation dynamics in mammalian cells. Nature Biotechnology 2011 29 436 442 10.1038/nbt.1861 21516085 PMC3114636

[B42] Shiroguchi K Jia TZ Sims PA Xie XS Digital RNA sequencing minimizes sequence-dependent bias and amplification noise with optimized single-molecule barcodes. Proceedings of the National Academy of Sciences, USA 2012 109 1347 1352 10.1073/pnas.1118018109 PMC3268301 22232676

[B43] Tang F Barbacioru C Nordman E Bao S Lee C Wang X Tuch BB Heard E Lao K Surani MA Deterministic and stochastic allele specific gene expression in single mouse blastomeres. PLoS ONE 2011 6 e21208 10.1371/journal.pone.0021208 21731673 PMC3121735

[B44] Miyanari Y Torres-Padilla M Control of ground-state pluripotency by allelic regulation of *Nanog*. Nature 2012 483 470 473 10.1038/nature10807 22327294

[B45] Turro E Su SY Goncalves A Coin LJ Richardson S Lewin A Haplotype and isoform specific expression estimation using multi-mapping RNA-seq reads. Genome Biology 2011 12 R13 10.1186/gb-2011-12-2-r13 21310039 PMC3188795

[B46] Anders S Huber W Differential expression analysis for sequence count data. Genome Biology 2010 11 R106 10.1186/gb-2010-11-10-r106 20979621 PMC3218662

[B47] Neal RM Slice sampling. The Annals of Statistics 2003 31 705 767 10.1214/aos/1056562461

[B48] Best DJ Rayner JCW Goodness of fit for the Poisson distribution. Statistics & Probability Letters 1999 44 259 265 10.1016/S0167-7152(99)00017-623606987

[B49] Core LJ Waterfall JJ Lis JT Nascent RNA sequencing reveals widespread pausing and divergent initiation at human promoters. Science 2008 322 1845 1848 10.1126/science.1162228 19056941 PMC2833333

