# Supplementary material for: Inferring the kinetics of stochastic gene expression from single-cell RNA-sequencing data
Source: Genome Biol. 2013 Jan 28;14(1):R7. doi: 10.1186/gb-2013-14-1-r7 (PMC3663116; doi:10.1186/gb-2013-14-1-r7)
Supplement: Additional file 1 — Supplemental methods and list of supplemental figures. [file gb-2013-14-1-r7-S1.PDF]

Supplementary information:
Inferring the kinetics of stochastic gene expression from single-cell RNA-sequencing data

Contents

Supplementary methods **2**
Deriving the master equation from a continuous time Markov chain 2

Reference **4**

Supplementary Figure 1 **7**

Supplementary Figure 2 **8**

Supplementary Figure 3 **9**

Supplementary Figure 4 **10**

Supplementary Figure 5 **11**

Supplementary Figure 6 **12**

Supplementary Figure 7 **13**

Supplementary Figure 8 **14**

Supplementary Figure 9 **15**

Supplementary Figure 10 **16**

Supplementary Figure 11 **17**

Supplementary Figure 12 **18**

Supplementary Figure 13 **19**

Supplementary Figure 14 **20**

Supplementary Figure 15 **21**

Supplementary Figure 16 **22**

Supplementary methods

Deriving the master equation from a continuous time Markov chain

In this section, we define the continuous time Markov chain that underpins the chemical master equations. This work was first described by Peccoud and Ycart (1995). We consider a continuous time Markov chain $W = \{W(t) = (Y(t), X(t))|t \in [0, \infty)\}$ with a state space

$$\mathcal{S} = \{(y, x)|y \in \{0, 1\}, x \in \{0, 1, 2, \ldots\}\},$$

where the first state y denotes the promoter status of a gene such that $y = 1$ if the gene is active and 0 otherwise. The second state, x, of the process represents the number of mRNA molecules of the gene. We assume that the process has stationary transition probabilities such that

$$P_{(y,x)\to(y',x')}(h) = P((Y(t+h) = y', X(t+h) = x')|(Y(t) = y, X(t) = x)),$$

where the transition probabilities are independent of t.

The stationary transition probabilities of the process are given by

$$\begin{aligned} P_{(1,x)\to(0,x)}(h) &= k_{off}h + o(h) && (1)\\ P_{(1,x)\to(1,x+1)}(h) &= sh + o(h) && (2)\\ P_{(1,x)\to(1,x-1)}(h) &= xdh + o(h) && (3)\\ P_{(0,x)\to(1,x)}(h) &= k_{on}h + o(h) && (4)\\ P_{(0,x)\to(0,x-1)}(h) &= xdh + o(h) && (5) \end{aligned}$$

Here $o(h)$ is any function such that $\lim_{h\downarrow 0} \frac{o(h)}{h} = 0$. Transitions between states not specified by the above equations are not allowed. The corresponding kinetic model for stochastic gene expression is illustrated in Figure 1A.

We describe the physical meanings of the kinetic parameters associated with the transition probabilities. Let $T_{y=1}$ denote the waiting time of a gene in an active state until it first jumps to an inactive state. The random variable $T_{y=1}$ follows an exponential distribution with parameter k_{off}, where the average waiting time in an active state is $\frac{1}{k_{off}}$. Similarly, we let the random variable $T_{y=0}$ denote the waiting time of a gene in an inactive state. This random variable is exponentially distributed with parameter k_{on}, and the average waiting time in the inactive state is $\frac{1}{k_{on}}$.

To interpret the decay rate d, we assume that each mRNA molecule degrades according to its own decay process $V = \{V(t) \in \{0,1\} | t \in [0,\infty)\}$, where $V(t) = 1$ if a mRNA molecule survives at time t or $V(t) = 0$ otherwise. The decay processes of mRNA molecules are assumed to be identical and independent. The process has the following stationary transition probability

$$P_{(1)\to(0)}(h) \;\; = \;\; dh + o(h).$$

Thus, the lifetime of a mRNA molecule follows an exponential distribution with parameter d, and the average lifetime of an mRNA molecule is $\frac{1}{d}$.

Finally, the kinetic parameter s is related to a Poisson process. Let $U = \{U(t) \in \{0,1,2,\ldots\} | t \in [0,\infty)\}$ be a Poisson process where $U(t)$ represents the total number of synthesized mRNA molecules of a gene in the time interval $[0,t]$ and we assume that the gene is always in the active state. The stationary transition probability is given by

$$P_{(x)\to(x+1)}(h) \;\; = \;\; sh + o(h).$$

It is well known that $U(t)$ follows a Poisson distribution with mean st. Therefore, the average number of mRNA molecules synthesized in time t is st. If we set $t = \frac{1}{k_{off}}$, we obtain the average number of synthesized mRNA molecules while a gene remains in an active state, $\frac{s}{k_{off}}$, which can be interpreted as the transcriptional efficiency. Finally, the inverse of the decay rate d denotes the average lifetime of an mRNA molecule, meaning that k_{on}, k_{off}, and s are all measured in units of $\frac{1}{t}$.

Peccoud and Ycart (1995) showed that if $P^y(x,t)$ denotes the probability of having x mRNA molecules of a gene at time t when in state y, then:

$$P^y(x,t+h) \;\; = \;\; \sum_{(y',x')} P^{y'}(x',t) P_{(y',x')\to(y,x)}(h).$$

When the gene is inactive ($y = 0$), we have

$$\begin{aligned}
P^0(x, t+h) &= P^1(x,t)P_{(1,x)\to(0,x)}(h) + P^0(x+1,t)P_{(0,x+1)\to(0,x)}(h) \\
&\quad + P^0(x,t)P_{(0,x)\to(0,x)}(h) \\
&= P^1(x,t)P_{(1,x)\to(0,x)}(h) + P^0(x+1,t)P_{(0,x+1)\to(0,x)}(h) \\
&\quad + P^0(x,t)(1 - P_{(0,x)\to(1,x)}(h) - P_{(0,x)\to(0,x-1)}(h)) \\
&= P^1(x,t)k_{off}h + P^0(x+1,t)(x+1)dh \\
&\quad + P^0(x,t)(1 - k_{on}h - dxh) + o(h).
\end{aligned}$$

Dividing by h and taking the limit as $h \downarrow 0$, we obtain

$$\frac{\partial P^0(x,t)}{\partial t} = k_{off}P^1(x,t) + d(x+1)P^0(x+1,t) - (k_{on} + dx)P^0(x,t)$$

Similarly, when the gene is active, we have

$$\begin{aligned}
\frac{\partial P^1(x,t)}{\partial t} &= k_{on}P^0(x,t) + sP^1(x-1,t) + d(x+1)P^1(x+1,t) \\
&\quad -(k_{off} + s + dx)P^1(x,t),
\end{aligned}$$

where $x \geq 1$. If $x = 0$, we have

$$\frac{\partial P^1(0,t)}{\partial t} = k_{on}P^0(0,t) + dP^1(1,t) - (k_{off} + s)P^1(0,t).$$

References

Peccoud, J. and Ycart, B., 1995. Markovian modelling of gene product synthesis. *Theoretical Population Biology*, **48**:222–234.

Supplementary Figure Legends

S1 The Poisson-Beta model is represented as a directed graphical model. 7

S2 Scatter plots showing the correlation of read counts between two technical replicates of mouse ES cells. Highly expressed genes above the cutoff (30 (S2A), 50 (S2B), and 100 (S2C)) are highlighted in red. 8

S3 Scatter plot showing the spread of $k_{on,i}$ and $k_{off,i}$ of each gene (12,551 genes). Red points mean non-identifiable genes. 9

S4 Scatter plots showing correlation between the parameter estimates and the true values (N=3, $\beta_{k_{on,i}} = \beta_{k_{off,i}} = 100$). s_i in (C) and (F) are multiplied by the size factor and the length of transcripts. 10

S5 Scatter plots showing correlation between the parameter estimates and the true values (N=6, $\beta_{k_{on,i}} = \beta_{k_{off,i}} = 100$). s_i in (C) and (F) are multiplied by the size factor and the length of transcripts. 11

S6 Scatter plots showing correlation between the parameter estimates and the true values (N=12, $\beta_{k_{on,i}} = \beta_{k_{off,i}} = 100$). s_i in (C) and (F) are multiplied by the size factor and the length of transcripts. 12

S7 Scatter plots showing correlation between the parameter estimates and the true values (N=20, $\beta_{k_{on,i}} = \beta_{k_{off,i}} = 100$). s_i in (C) and (F) are multiplied by the size factor and the length of transcripts. 13

S8 Scatter plots showing correlation between the parameter estimates and the true values (N=100, $\beta_{k_{on,i}} = \beta_{k_{off,i}} = 100$). s_i in (C) and (F) are multiplied by the size factor and the length of transcripts. 14

S9 Scatter plot showing the effect of priors on $k_{on,i}$ and $k_{off,i}$. We used the following priors: $\alpha_{k_{on,i}} = 1$, $\beta_{k_{on,i}} = 10$, $\alpha_{k_{off,i}} = 1$, $\beta_{k_{off,i}} = 10$ for (A) and $\alpha_{k_{on,i}} = 1$, $\beta_{k_{on,i}} = 10000$, $\alpha_{k_{off,i}} = 1$, $\beta_{k_{off,i}} = 10000$ for (B). 15

S10 Scatter plot showing the effect of priors on parameter estimates of $k_{on,i}$ and $k_{off,i}$. 16

S11 Scatter plots showing correlation between the parameter estimates and the true values ($k_{on,i}$ is small and $k_{off,i}$ is large, N=100, $\beta_{k_{on,i}} = \beta_{k_{off,i}} = 100$). Each red point represents non-identifiable genes with relatively large values of $k_{off,i}$ and low values of $k_{on,i}$. s_i in (C) and (F) are multiplied by the size factor and the length of transcripts. 17

S12 Scatter plots showing correlation between the parameter estimates and the true values ($k_{on,i}$ is large and $k_{off,i}$ is small, N=100, $\beta_{k_{on,i}} = \beta_{k_{off,i}} = 100$). Each red point represents non-identifiable genes with relatively large values of $k_{on,i}$ and low values of $k_{off,i}$. s_i in (C) and (F) are multiplied by the size factor and the length of transcripts. 18
S13 Correlation of the average expression level with other promoter kinetic parameters. In all the panels, the average expression level is plotted on the y-axis. ρ is the Spearman correlation coefficient. 19
S14 Correlation of transcriptional kinetics with RNA polymerase II binding in mouse ES cells. The average expression level is plotted on the x-axis. In all the panels, the following are plotted on the y-axis: the gene body activity (A), the gene promoter activity (B), and the pause index (C). 20
S15 Correlation of transcriptional kinetics with RNA polymerase II binding in mouse ES cells. The cutoff used to filter out lowly expressed genes is 100. 21
S16 Correlation of the average expression level with histone modifications in mouse ES cells. (A) Box plots that compare the average expression level (A) in the four groups. The H3K4me3 group is significantly different from the others: $P < 10^{-16}$ for $E(x)$ by Mann-Whitney U-test. (D) The profiles of H3K4me3, H3K27me3, and H3K36me3 ChIP-seq reads mapped near TSSs are shown for genes with values of the three kinetic quantities in the top 10% (red), middle 10% (green), and bottom 10% (blue) of the relevant distribution. The y-axis is the total number of reads mapped to each position. 22

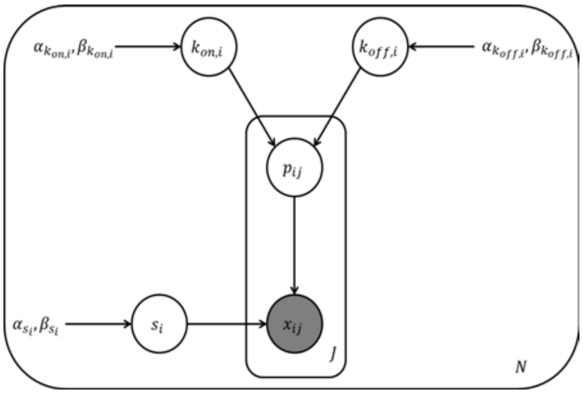
$\alpha_{k_{on,i}}, \beta_{k_{on,i}}$
$k_{on,i}$
$k_{off,i}$
$\alpha_{k_{off,i}}, \beta_{k_{off,i}}$
p_{ij}
$\alpha_{s_i}, \beta_{s_i}$
s_i
x_{ij}
J
N

Figure S1

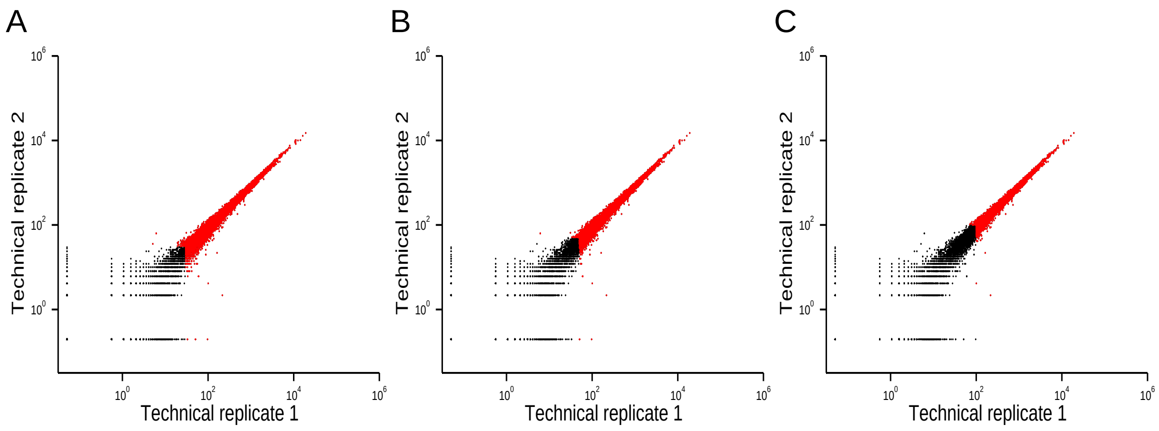

Figure S2

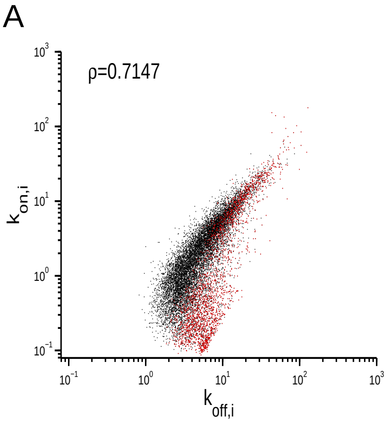

Figure S3

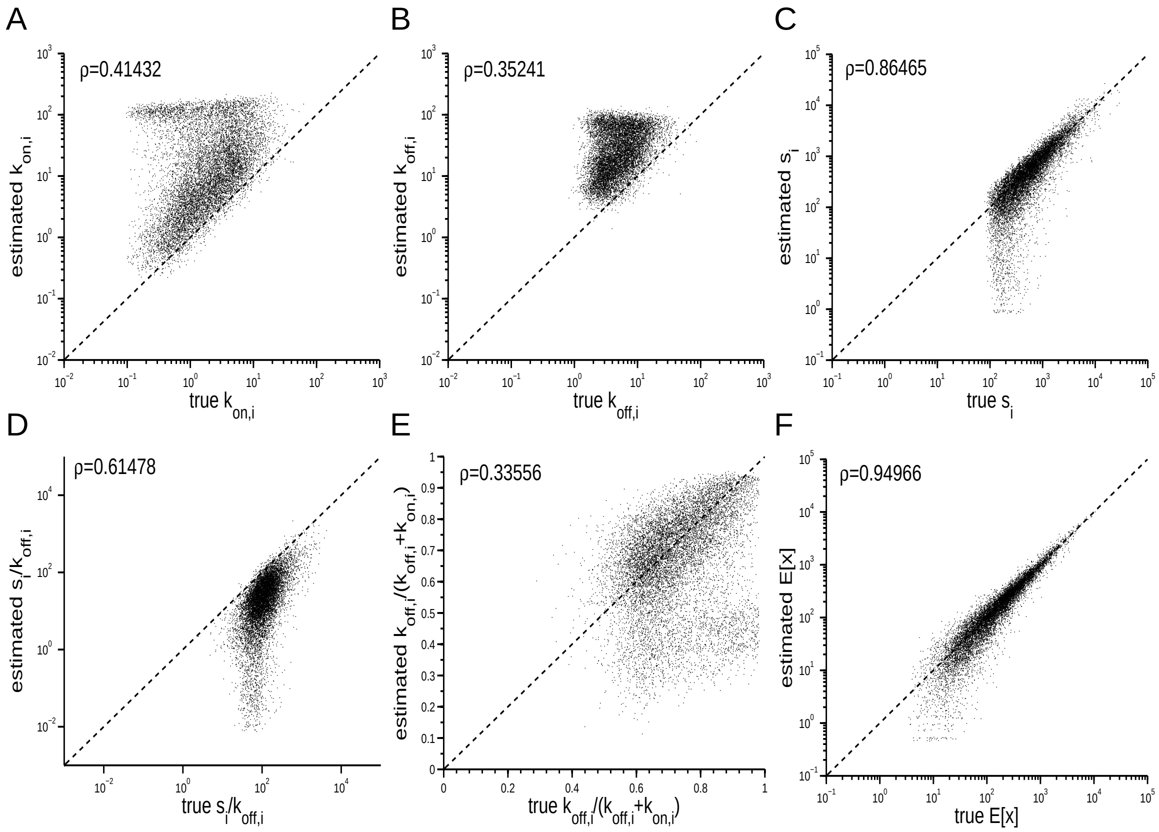
A
ρ=0.41432
estimated k_on,i
true k_on,i
B
ρ=0.35241
estimated k_off,i
true k_off,i
C
ρ=0.86465
estimated s_i
true s_i
D
ρ=0.61478
estimated s_i/k_off,i
true s_i/k_off,i
E
ρ=0.33556
estimated k_off,i/(k_off,i+k_on,i)
true k_off,i/(k_off,i+k_on,i)
F
ρ=0.94966
estimated E[x]
true E[x]

Figure S4

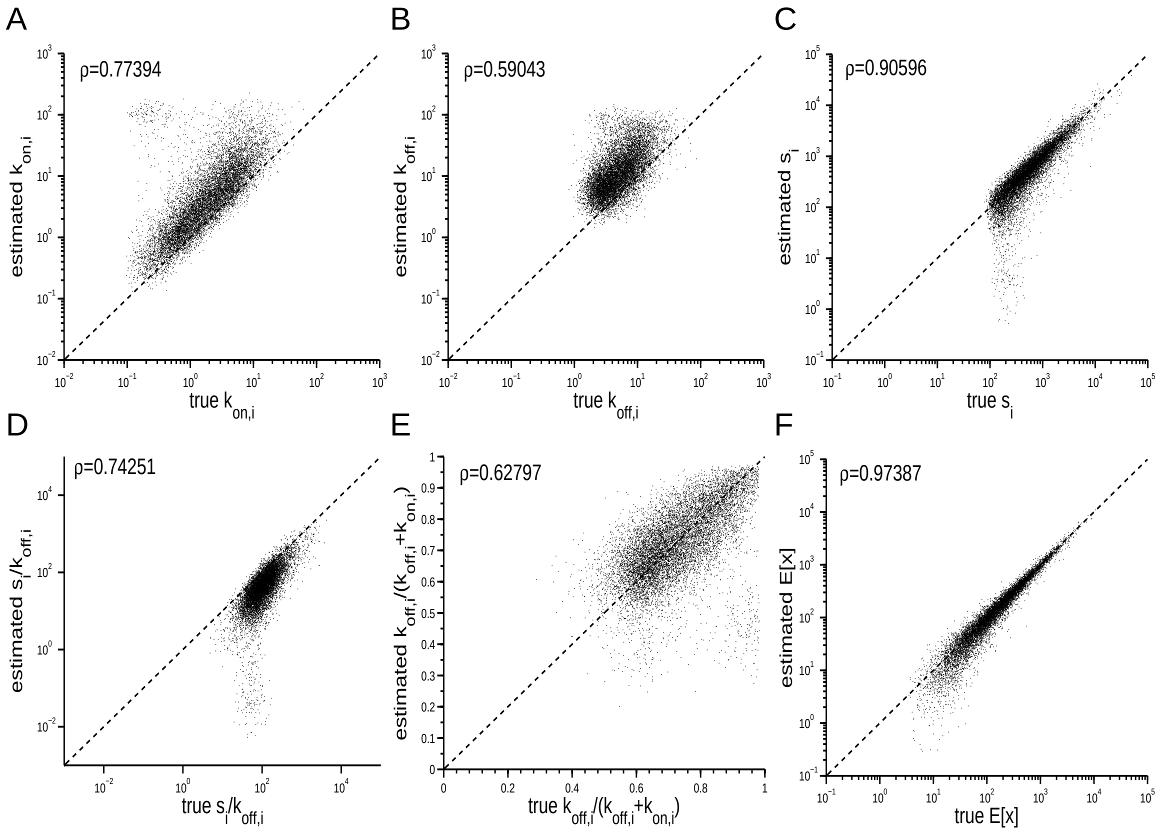
A
ρ=0.77394
estimated k_on,i
true k_on,i
B
ρ=0.59043
estimated k_off,i
true k_off,i
C
ρ=0.90596
estimated s_i
true s_i
D
ρ=0.74251
estimated s_i/k_off,i
true s_i/k_off,i
E
ρ=0.62797
estimated k_off,i/(k_off,i+k_on,i)
true k_off,i/(k_off,i+k_on,i)
F
ρ=0.97387
estimated E[x]
true E[x]

Figure S5

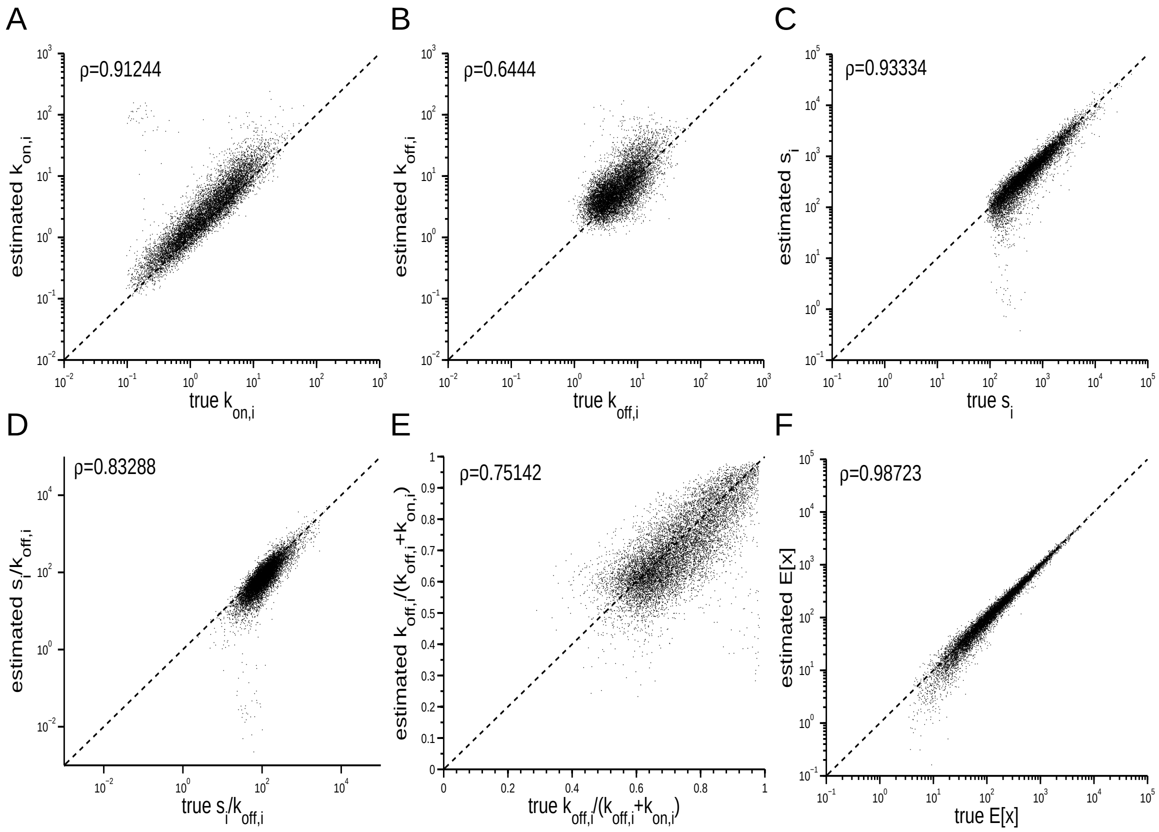
A
ρ=0.91244
estimated k_on,i
true k_on,i
B
ρ=0.6444
estimated k_off,i
true k_off,i
C
ρ=0.93334
estimated s_i
true s_i
D
ρ=0.83288
estimated s_i/k_off,i
true s_i/k_off,i
E
ρ=0.75142
estimated k_off,i/(k_off,i+k_on,i)
true k_off,i/(k_off,i+k_on,i)
F
ρ=0.98723
estimated E[x]
true E[x]

Figure S6

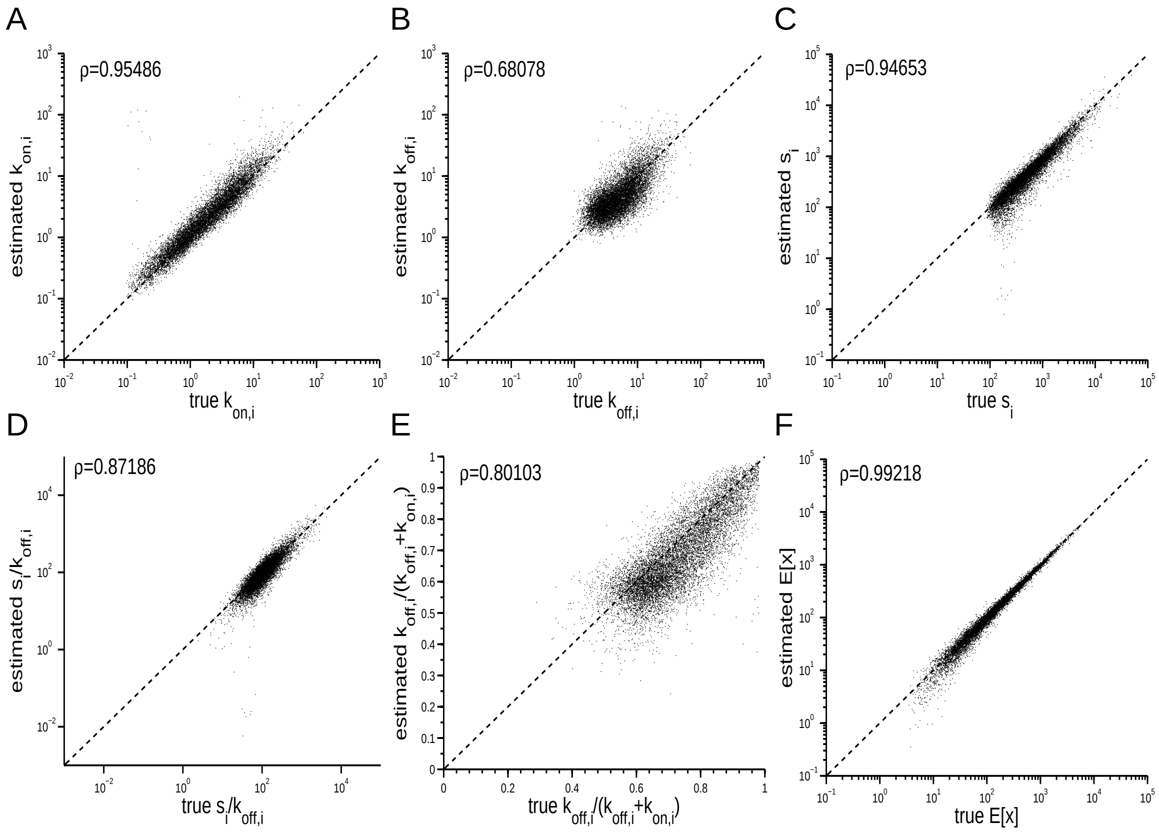
A
ρ=0.95486
estimated k_on,i
true k_on,i
B
ρ=0.68078
estimated k_off,i
true k_off,i
C
ρ=0.94653
estimated s_i
true s_i
D
ρ=0.87186
estimated s_i/k_off,i
true s_i/k_off,i
E
ρ=0.80103
estimated k_off,i/(k_off,i+k_on,i)
true k_off,i/(k_off,i+k_on,i)
F
ρ=0.99218
estimated E[x]
true E[x]

Figure S7

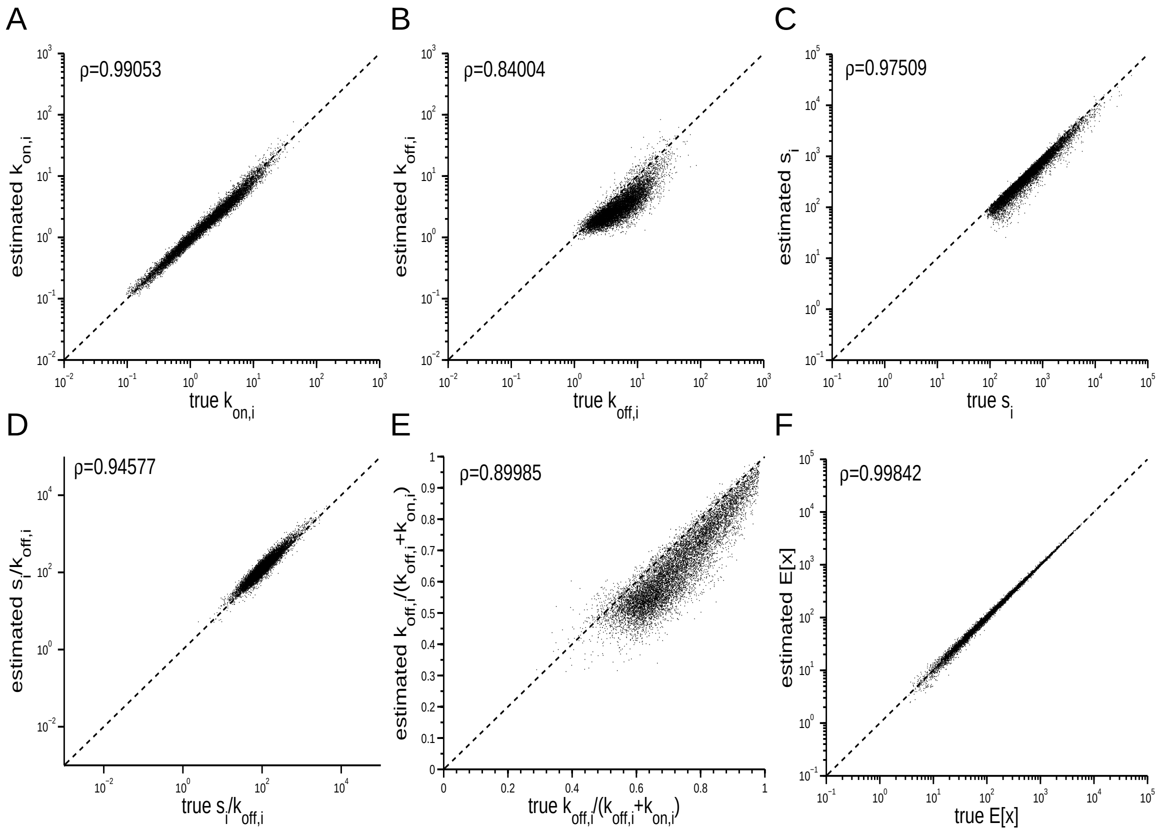
A
ρ=0.99053
estimated k_on,i
true k_on,i
B
ρ=0.84004
estimated k_off,i
true k_off,i
C
ρ=0.97509
estimated s_i
true s_i
D
ρ=0.94577
estimated s_i/k_off,i
true s_i/k_off,i
E
ρ=0.89985
estimated k_off,i/(k_off,i+k_on,i)
true k_off,i/(k_off,i+k_on,i)
F
ρ=0.99842
estimated E[x]
true E[x]

Figure S8

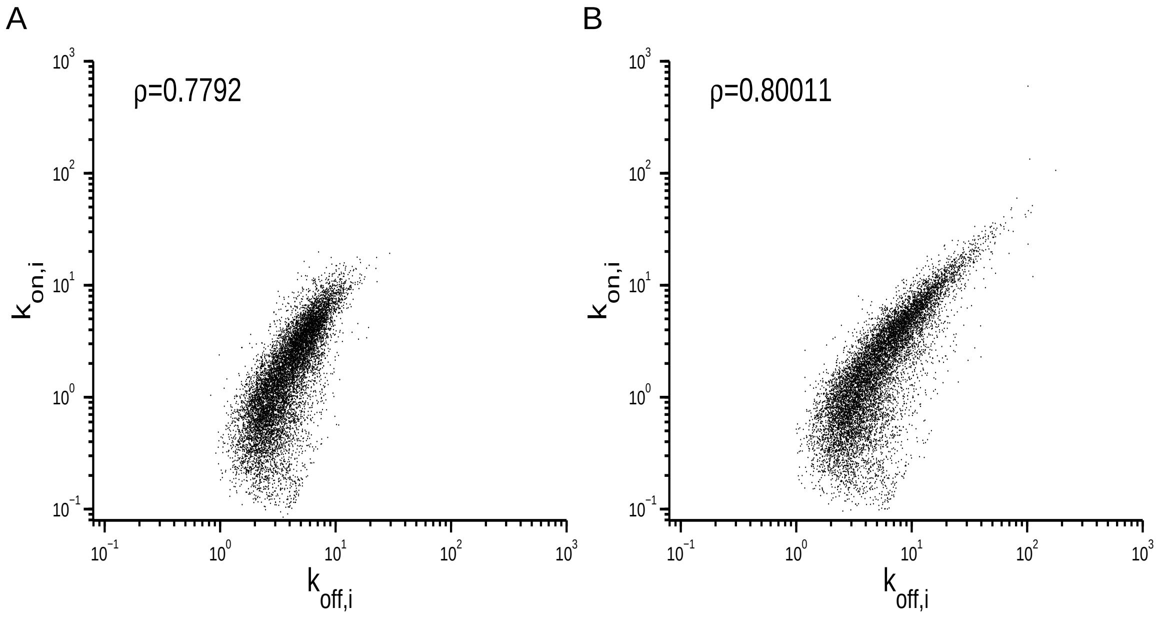

Figure S9

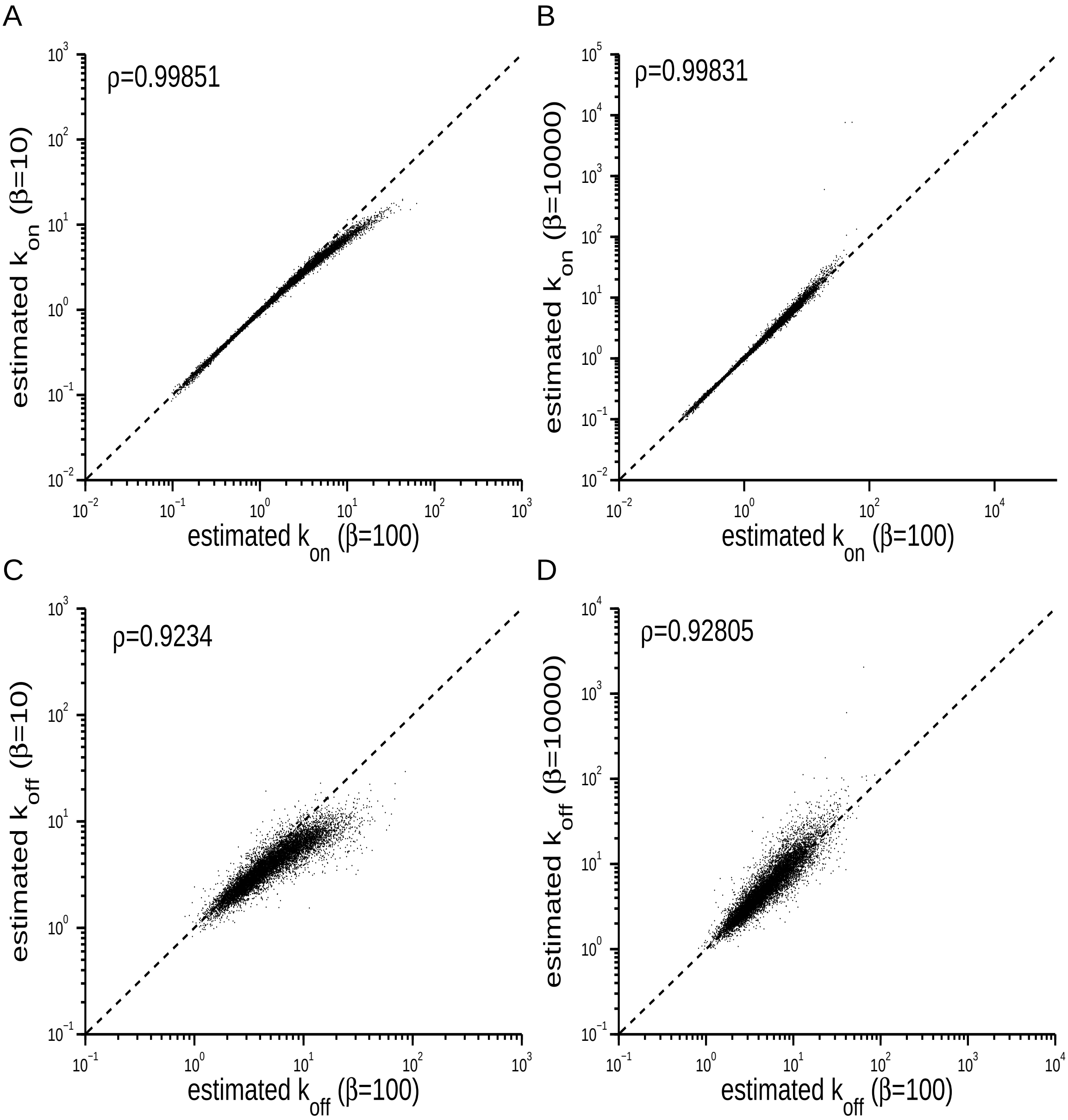
A
ρ=0.99851
estimated k_{on} (β=10)
estimated k_{on} (β=100)
B
ρ=0.99831
estimated k_{on} (β=10000)
estimated k_{on} (β=100)
C
ρ=0.9234
estimated k_{off} (β=10)
estimated k_{off} (β=100)
D
ρ=0.92805
estimated k_{off} (β=10000)
estimated k_{off} (β=100)

Figure S10

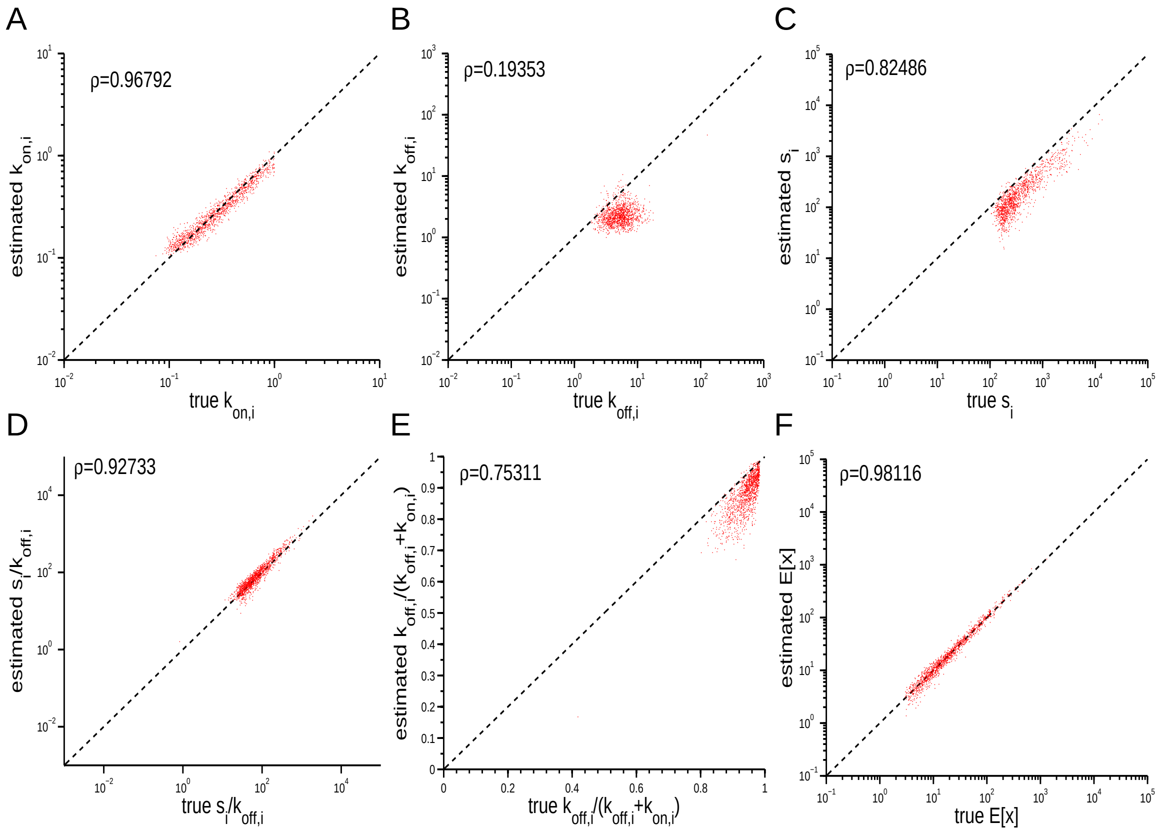
A
ρ=0.96792
estimated k_on,i
true k_on,i
B
ρ=0.19353
estimated k_off,i
true k_off,i
C
ρ=0.82486
estimated s_i
true s_i
D
ρ=0.92733
estimated s_i/k_off,i
true s_i/k_off,i
E
ρ=0.75311
estimated k_off,i/(k_off,i+k_on,i)
true k_off,i/(k_off,i+k_on,i)
F
ρ=0.98116
estimated E[x]
true E[x]

Figure S11

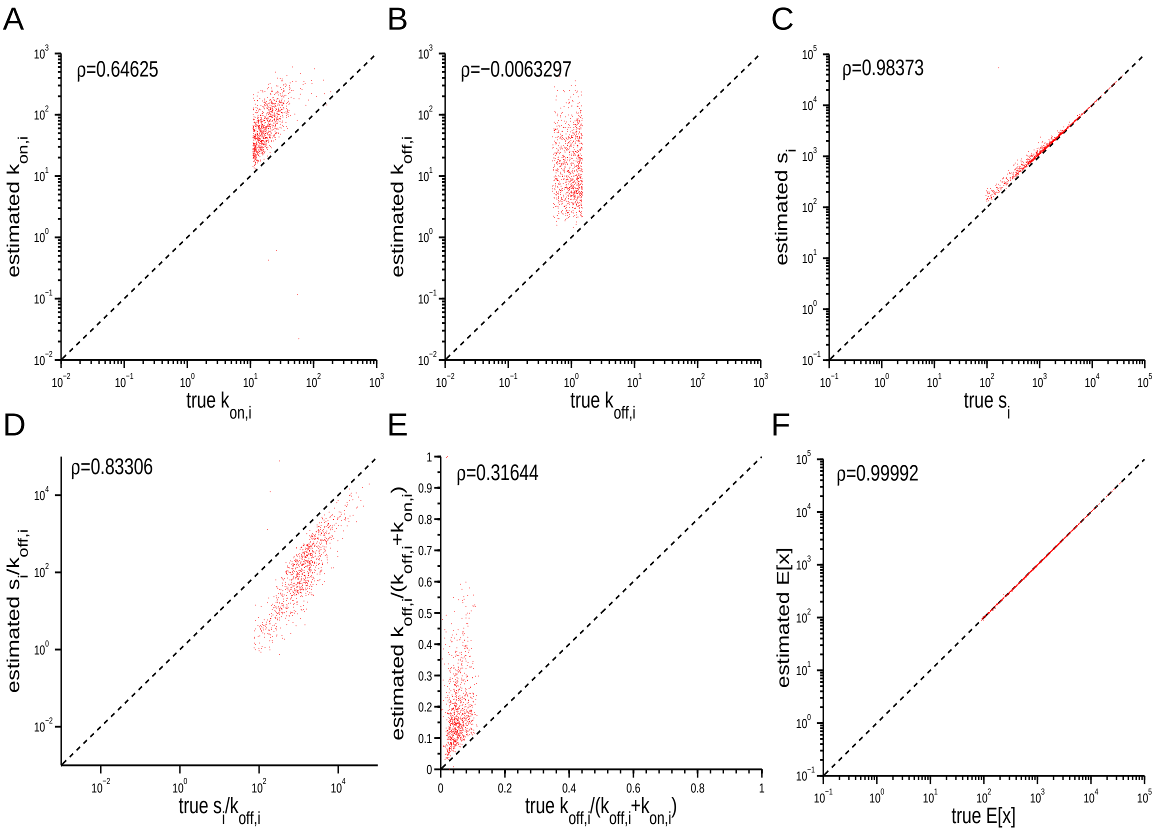
A
ρ=0.64625
estimated k_on,i
true k_on,i
B
ρ=−0.0063297
estimated k_off,i
true k_off,i
C
ρ=0.98373
estimated s_i
true s_i
D
ρ=0.83306
estimated s_i/k_off,i
true s_i/k_off,i
E
ρ=0.31644
estimated k_off,i/(k_off,i+k_on,i)
true k_off,i/(k_off,i+k_on,i)
F
ρ=0.99992
estimated E[x]
true E[x]

Figure S12

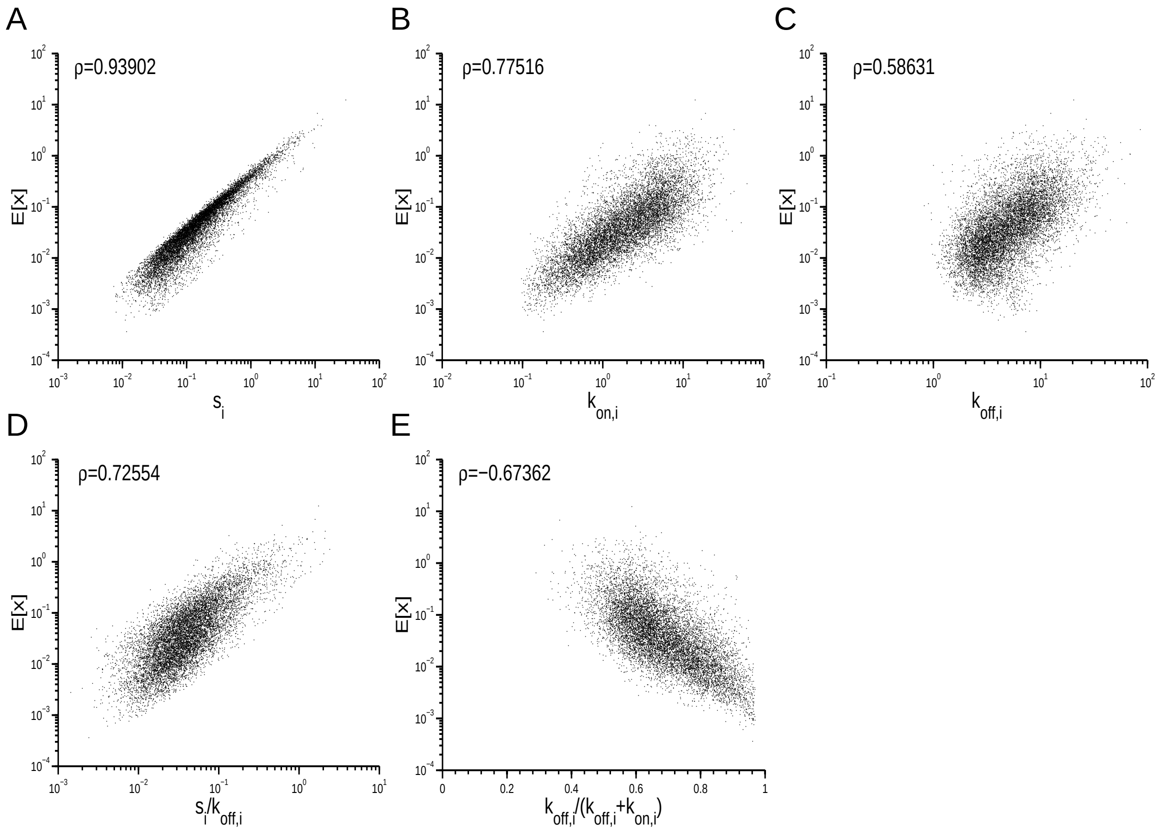
A
ρ=0.93902
E[x]
s_i
B
ρ=0.77516
E[x]
$k_{on,i}$
C
ρ=0.58631
E[x]
$k_{off,i}$
D
ρ=0.72554
E[x]
$s_i/k_{off,i}$
E
ρ=−0.67362
E[x]
$k_{off,i}/(k_{off,i}+k_{on,i})$

Figure S13

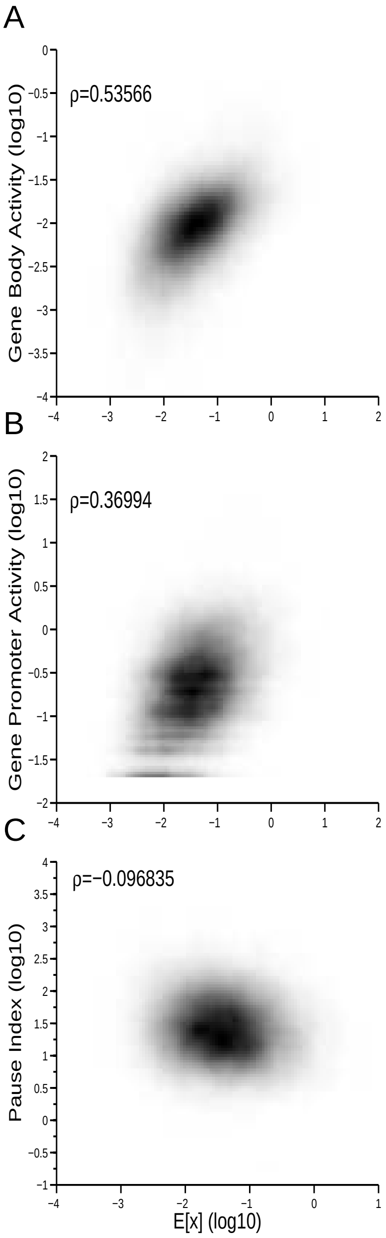
A
ρ=0.53566
Gene Body Activity (log10)
B
ρ=0.36994
Gene Promoter Activity (log10)
C
ρ=−0.096835
Pause Index (log10)
E[x] (log10)

Figure S14

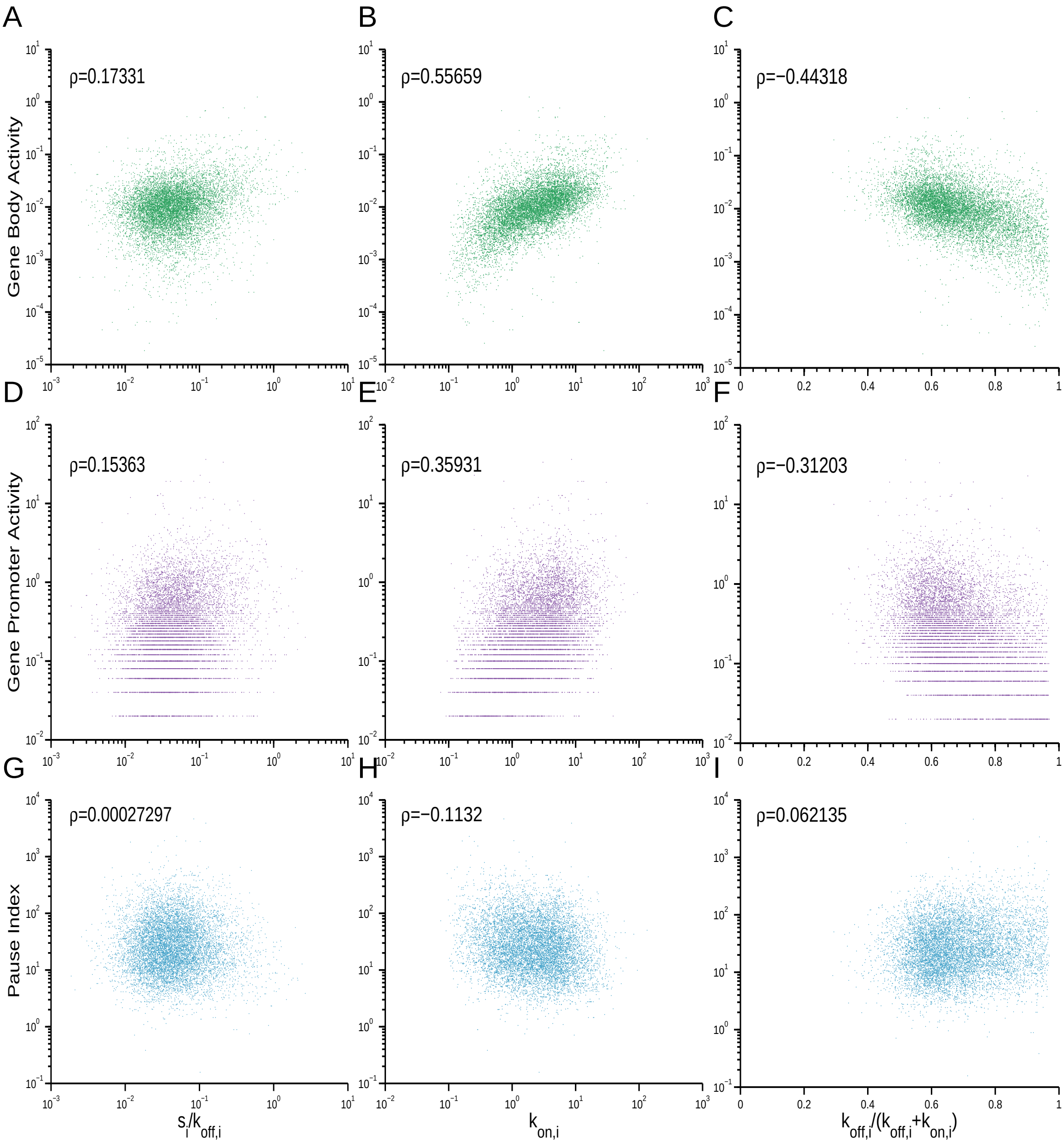

A
B
C
D
E
F
G
H
I
Gene Body Activity
Gene Promoter Activity
Pause Index
ρ=0.17331
ρ=0.55659
ρ=−0.44318
ρ=0.15363
ρ=0.35931
ρ=−0.31203
ρ=0.00027297
ρ=−0.1132
ρ=0.062135
s_i/k_off,i
k_on,i
k_off,i/(k_off,i+k_on,i)

Figure S15

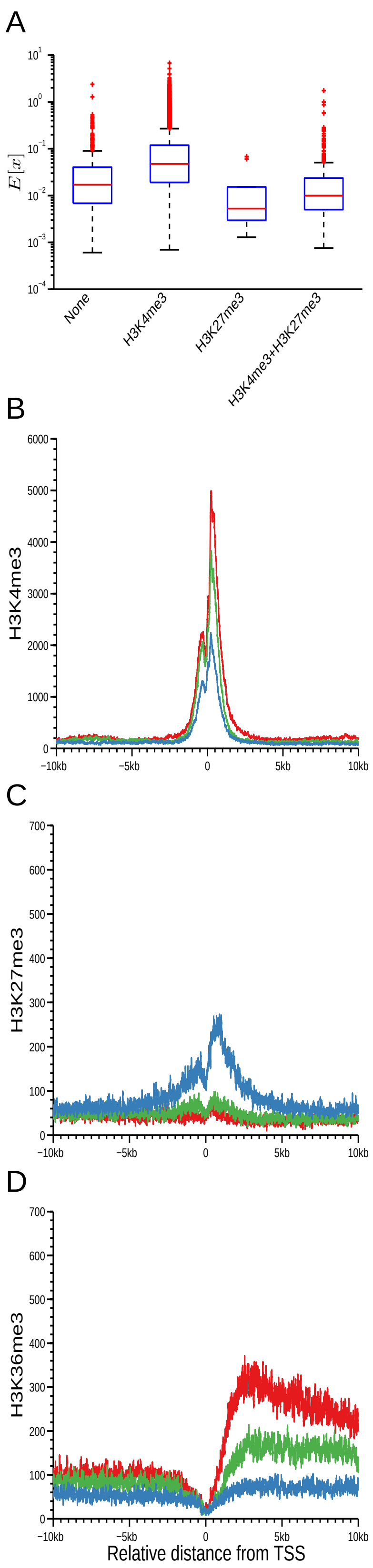
A
$E[x]$
None
H3K4me3
H3K27me3
H3K4me3+H3K27me3
B
H3K4me3
C
H3K27me3
D
H3K36me3
−10kb
−5kb
0
5kb
10kb
Relative distance from TSS

Figure S16
